# A Comparison of Lipid Contents in Different Types of Peanut Cultivars Using UPLC-Q-TOF-MS-Based Lipidomic Study

**DOI:** 10.3390/foods11010004

**Published:** 2021-12-21

**Authors:** Yuting Huang, Rui Ma, Yongju Xu, Kai Zhong, Qian Bu, Hong Gao

**Affiliations:** 1 College of Biomass Science and Engineering, Sichuan University, Chengdu 610065, China; hyt8085@163.com (Y.H.); marui8003@163.com (R.M.); eric211@163.com (K.Z.); 2 Industrial Crops Research Institute Sichuan Academy of Agricultural Sciences, Chengdu 610300, China; xyj2002024@163.com; 3 West China School of Public Health, Sichuan University, Chengdu 610065, China; buqian7978@scu.edu.cn

**Keywords:** peanuts, oleic acid, lipidomics, oxidation stability

## Abstract

Peanuts are a rich dietary source of lipids, which are essential for human health. In this study, the lipid contents of 13 peanut cultivars were analyzed using UPLC-Q-TOF-MS and GC–MS. The OXITEST reactor was used to test their lipid oxidation stabilities. A total of 27 subclasses, 229 individual lipids were detected. The combined analysis of lipid and oxidation stability showed that lipid unsaturation was inversely correlated with oxidation stability. Moreover, lipid profiles differed significantly among the different peanut cultivars. A total of 11 lipid molecules (TG 18:2/18:2/18:2, TG 24:0/18:2/18:3, TG 20:5/14:1/18:2, TG 18:2/14:1/18:2, PE 17:0/18:2, BisMePA 18:2/18:2, PG 38:5, PMe 18:1/18:1, PC 18:1/18:1, MGDG 18:1/18:1, TG 10:0/10:1/18:1) might be employed as possible indicators to identify high oleic acid (OA) and non-high OA peanut cultivars, based on the PLS-DA result of lipid molecules with a VIP value greater than 2. This comprehensive analysis will help in the rational selection and application of peanut cultivars.

## 1. Introduction

The peanut (*Arachis hypogaea* L.) is one of the most widely grown oilseed crops in the world. It is rich in lipids, proteins, and amino acids, and it is used for producing peanut oil and peanut butter [1,2,3,4]. Due to the commitment to eating healthy food, many cultivars of peanuts, especially those with high oleic acid (OA), have been developed. Consumption of high OA peanuts, which are (proven) highly beneficial antioxidants, may help reduce oxidative stress-induced disorders. Current evidence supports the positive link between OA and health management of gastric injury [5], myocardial injury, cardiovascular insulin resistance, and endothelial dysfunction [6]. However, a comparative analysis on the quality of different peanut cultivars is lacking at present. Therefore, it is vital to determine the quality of different cultivars in light of the increasing number of high-OA peanut cultivars and other functional peanuts.

In recent years, researchers have paid increasing attention to food quality analysis, using different analytical methods. Initially, the quality of oil crops was determined by oil content [7], saponification value, and acid value [8]. These methods simply assess the quality of oil crops; hence, they are limited, as they do not include compositional analysis. Subsequently, fatty acid composition analysis was widely adopted to assess food quality. However, this method cannot be used to analyze single lipid species. In this study, lipidomics, a relatively new technique, was chosen to investigate the qualitative and quantitative profile of intact lipid molecules in the study samples [9,10]. Q Exactive is a recently developed technique with extremely high resolution, sensitivity, and mass accuracy. It combines the ion filtration of a highly selective quadrupole with Orbitrap high-resolution accurate mass number (HR/AM) measurement technology to exhibit strong power for fragment ion scanning [11]. Hao Liu et al. researched the dynamic changes of lipid molecular species in high-OA seed and the global gene expression profile of peanut cultivars at six time points during seed development [12,13]. Rabiatu Bonku et al. analyzed the health aspects of peanuts as outcomes of their chemical compositions [14]. It is necessary to compare the lipid contents between high-OA and non-high OA peanuts.

The oxidative stability of lipids is one of the most important properties of oil crop processing products. It influences the flavor, nutrition, and shelf life. Presently, several techniques for measuring the oxidation state of these oil crop-processing products have been developed, such as differential scanning calorimetry (DSC) [15], pressure DSC (PDSC) [16], and electron spin resonance (ESR) [17]. The OXITEST reactor (Velp Scientifica, Usmate, Milan, Italy) can be used for the oxidative stability analysis. It is an instrument that subjects a sample to high oxidative stress (high temperature and oxygen overpressure) and evaluates the oxidative state of the sample in a short time. The OXITEST reactor is reliable and can directly analyze solid, liquid, and doughy foods without needing fat portions to be isolated first [18].

The main aim of the present study was to analyze and compare the lipid composition and oxidation stability of 13 different peanut cultivars. We used lipidomics and GC–MS absolute quantification to compare and analyze the lipids. In addition, the OXITEST analysis was tested to identify and compare the oxidative stability of these peanut cultivars. It is important to note that a joint analysis of lipid and oxidative stability was also performed to identify the potential association between lipid structure and oxidative stability of peanuts. Clarification of the information is likely to have significant implications for production, storage, nutrition selection, and economic benefits of oil seeds.

## 2. Materials and Methods

### 2.1. Plant Materials and Chemicals

**Figure 1:**
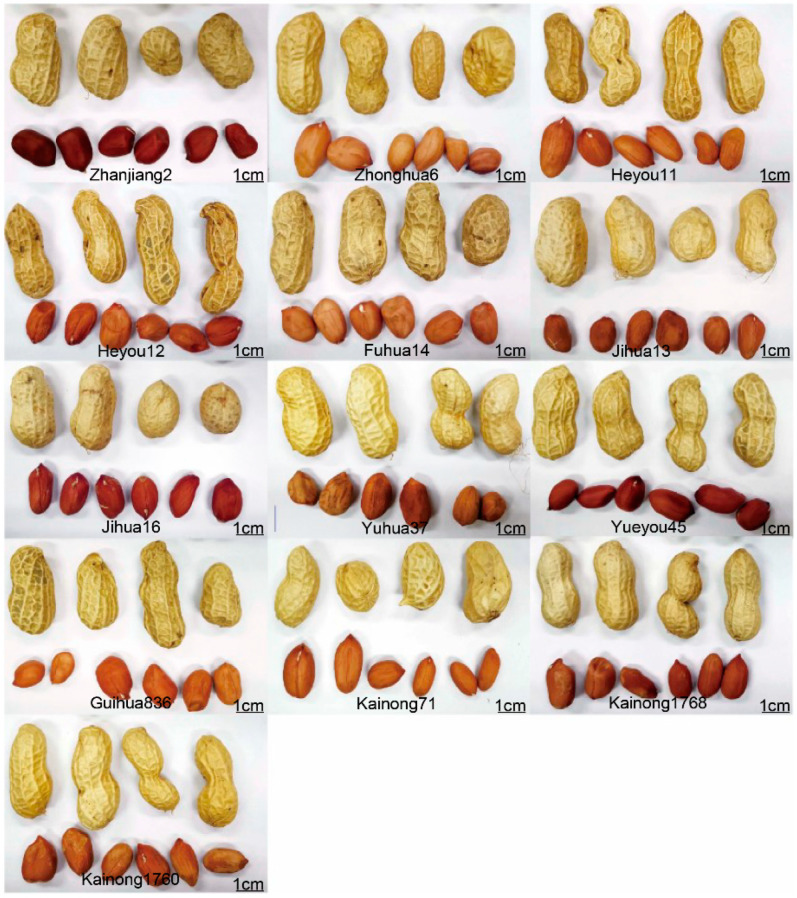
Phenotypes of 13 peanut cultivars.

Thirteen peanut cultivars used in the study were provided by the Industrial Crops Research Institute Sichuan Academy of Agricultural Sciences. The cultivars were grown in the Jintang County, Chengdu City experimental field (Sichuan Province, China) for four months under normal growth conditions (Figure 1). The peanut cultivars used are Zhanjinag2, Zhonghua6, Heyuo11, Heyou12, Fuhua14, Jihua13, Jihua16, Yuhua37, Yueyou45, Guihua836, Kainong71, Kainong1768, and Kainong1760. At harvest, the nuts were peeled, shelled, ground into powder, and stored at −80 °C until use.

High-performance liquid chromatography (HPLC) grade methanol, acetonitrile, isopropanol, ammonium acetate, methyl-tert-butyl ether (MTBE) were purchased from Sigma-Aldrich (Saint Louis, MI, USA). Potassium hydroxide, n-hexane, trichloromethane, pentadecanoic acid were purchased from Aladdin (Shanghai, China).

### 2.2. GC–MS Analysis and Identification of the Fatty Acid Methyl Esters

The GC–MS analysis was tested via a reported method with some modifications [19,20]. The peanut powder (0.5 g) was placed in a falcon tube containing 5 mL n-hexane. Pentadecanoic acid was added as an internal standard and whirled for 30 s. Approximately 2 mL of 5 mol/L potassium hydroxide-methanol solution was added, spun for 1 min, and then centrifuged for 10 min. The supernatant was transferred to a new tube, and an equal volume of sterile distilled water was added to clean the extract. After centrifugation (10,000 rpm, 15 min), the supernatant was filtered through a membrane (pore size 0.22 μm) before analysis. A 1 μL aliquot of the sample was injected into SH-Rxi-5Sil MS capillary column (Shimadzu, Kyoto, Japan) using a 25:1 split injection port. The GC injector, ion source, transfer line, and detector temperatures were 280 °C, 230 °C, 235 °C, and 230 °C, respectively. The oven temperature was programmed as follows: 170 °C for 2 min, 220 °C at a rate of 20 °C/min, 240 °C at a rate of 50 °C/min, 240 °C for 2 min, 280 °C at a rate of 10 °C/min, 280 °C for 5 min. Each analysis took 18 min. The fatty acid methyl esters (FAMEs) were identified using the standard Supelco 37 Component FAME Mix (Supelco, Darmstadt, Germany) and a mass spectra database search (NIST 14 Library). The concentration of the FAMEs was calculated according to the ratio of different FAMEs peak areas with the internal standard (15:0) peak area. The fatty acid composition is expressed as absolute concentrations (mg/g peanut). The analyses were performed in triplicates.

### 2.3. OXITEST Analysis

OXITEST analysis was performed using an OXITEST reactor (Velp Scientifica, Milan, Italy) according to a published report [21]. Briefly, 15 g peanuts power was spread evenly on two-sample dishes in a chamber. The temperature of the chamber was set at 90 °C, and the initial oxygen pressure was 6.0 bar. When the reaction time exceeds 200 h or the oxygen pressure is less than 1.5 bar, the monitor automatically stops. The induction periods (IPs), expressed in minutes, were obtained using the two-tangent method that is a method to obtain the intersection of tangent lines of oxidation curves of oxidation stable period and oxidation induction period. The longer the IP, the higher the stability against oxidation over time.

### 2.4. Untargeted Lipidomics Analysis

#### 2.4.1. Lipid Extraction

Total lipids were extracted using the Linhong Jiang method [22,23]. Methanol (150 μL) and MTBE (450 μL) were added to 35.0 mg peanuts powder in a 2 mL tissue grinding tube containing six clean mill beads. After high-speed shaking, 300 μL of 25% methanol/H_2_O solution was added and sonicated for 10 min. The mixture was centrifuged at 11,000 rpm for 10 min, and the organic phase was dried under a stream of nitrogen gas and stored at −80 °C.

#### 2.4.2. Instruments and Methods

For UPLC-Q-TOF-MS analysis, the dried samples were redissolved in 400 μL acetonitrile/ isopropanol/H_2_O (6.5/3.0/0.5, *v/v/v*). The 100 μL of the suspension was then transferred to insert pipes (Agilent, Santa Clara, America). The analysis was carried out in a UPLC system (Acquity, Waters, Worcester, America) equipped with an ACQUITY UPLC HSS T3 column (1.8 μm, 2.1 × 100 mm; Waters, Worcester, America) and a Xevo G2-S Q-TOF mass spectrometer (Waters, Worcester, America). The flow rate was 0.4 mL/min. Mobile phase A was made up of acetonitrile/water (40:60, *v/v*), while mobile phase B was acetonitrile/isopropanol (10:90, *v/v*), with both containing 10 mM ammonium acetate. The elution gradient was as follows: 0–3 min, 40–70% B; 3–15.5 min, 70–95% B; 15.5–19 min, 40% B. The column temperature was maintained at 55 °C, while the sample chamber temperature was maintained at 7 °C, the injection volume was 5 μL. Both positive and negative ion modes were used in the mass spectrometric analysis. The mass spectrometry parameters were set as follows: source temperature, 120 °C; the capillary voltage, 2.0 kV; cone gas, 50 L/h; desolation gas, 900 L/h at 550 °C; sampling cone voltage, 30 kV; the MS scan range, 50–1200 Da in full and all ion fragmentation scan mode.

#### 2.4.3. Data Processing and Analysis

Data analysis and lipid identification were performed using LipidSearch (version 4.2, Thermo Fisher Scientific, Waltham, MA, USA). First, the original data was transferred to DataExplorer, and the peaks were determined using precursor tolerance (±5 ppm), product tolerance (±5 ppm), and general database. Next, the data was aligned with the peaks, and the peak intensities were corrected as follows: *t*-score < 0.5, area score ≥ 0.7, duplication rank = 1, APValue < 0.05. ID quality filters were selected as A, B, and C grades. All of the lipid annotations generated by the LipidSearch software were manually verified by comparing molecular compositions, retention times (RTs), and specific fragmentation behaviors to lipid standards from the LIPID MAPS free database (http://www.lipidmaps.org (accessed on 10 July 2020)).

### 2.5. Statistical Analysis

The mean value, standard deviation, and significant level were calculated using the Microsoft Excel 2016 software and IBM SPSS Statistics 24.0 software (SPSS Inc., Chicago, IL, USA). The independent sample *t*-test was used to test the differences on lipids levels between high-OA and non-high OA peanuts at *p* < 0.05. SIMCA 13.0 software (Umetrics Inc., Malmo, Sweden) was used to perform principal component analysis (PCA), partial least squares discrimination analysis (PLS-DA), and the variable importance in projection (VIP) for the significantly changed lipids (VIP > 1, 2). As the classical linear dimensionality reduction technique, PCA (unsupervised) and PLS-DA (supervised) aim to obtain the most compact representations of the high-dimensional data under the sense of least square reconstruction error [24,25]. The GraphPad Prism 7.0 software (GraphPad Software Inc., San Diego, CA, USA), the Origin 2019b software (OriginLab Inc., Northampton, MA, USA) and the TBtools 1.075 software [26] were used to plot graphs.

## 3. Results

### 3.1. Identification and Quantification of Fatty Acids

**Figure 2:**
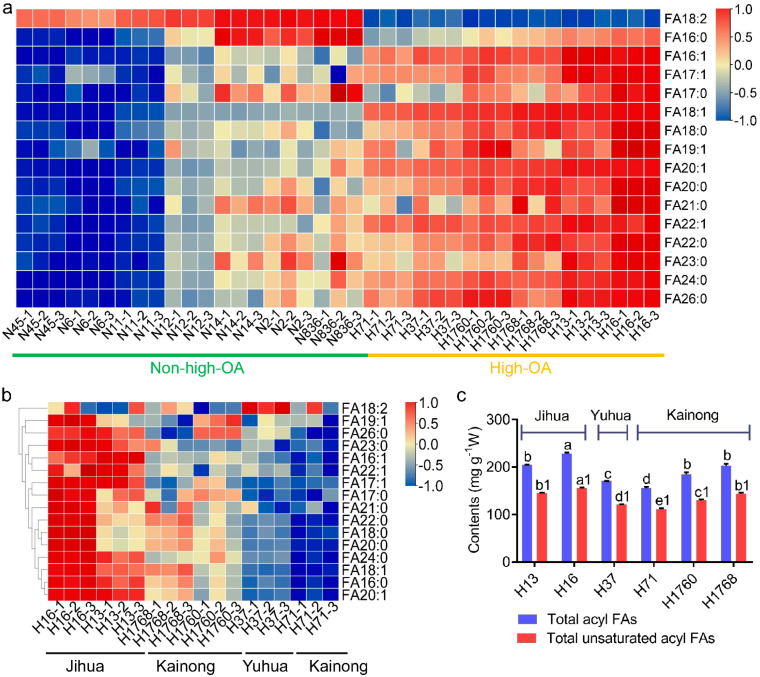
Fatty acid (FA) profiles of 13 peanut cultivars. (**a**) Heatmap of the FA content in high-OA peanut cultivars and non-high-OA peanut cultivars. Red (the scale of 1) and blue (the scale of −1) indicate increased and decreased levels, respectively. (**b**) Heatmap showing the content difference in FA in six high-OA peanut cultivars. (**c**) The content of total acyl FAs and total unsaturated acyl FAs of high-OA peanut cultivars. OA, oleic acid.

GC–MS analysis was performed to understand the composition of free fatty acids in different peanut cultivars. Here, 16 acyl fatty acids were characterized in the lipid profile (Table S1), and C19 had the highest diversity of molecular species. The OA content of Jihua16, Jihua13, Kainong1768, Kainong1760, Yuhua37, and Kainong71 exceeded 100 mg/g peanut powder, accounting for 66.43% of the total fatty acids. However, the OA content of Zhanjiang2, Zhonghua6, Heyou11, Heyou12, Fuhua14, Yueyou45, and Guihua836 was below 45 mg/g peanut powder, accounting for 26.40% of total fatty acids. For further analysis, the cultivars were divided into two groups, high and non-high OA, due to the significant difference in their OA contents. The linoleic acid contents of the high and non-high OA groups accounted for 66.43 and 42.14% of the total fatty acids. A circular heatmap showed that the high-OA group clustered outside while the non-high-OA group clustered inside the heatmap. These results indicate that the high-OA peanut group accumulated more of the 14 fatty acids, except linoleic and palmitic acid (Figure 2a). The high-OA group with better lipid performance was selected for further inter-group comparisons.

The high-OA group had six sub-cultivars (H13, H16, H37, H71, H1760, and H1768) from three cultivar series (Jihua, Yuhua, Kainong). A heatmap of the high-OA group showed that H16 (Jihua), H13 (Jihua), and H1768 (Kainong) had abundant fatty acids, while the fatty acid levels in H71 (Kainong) and H37 (Yuhua) were relatively low (Figure 2b). The samples ordered as H16 (Jihua) > H13 (Jihua) > H1768 (Kainong) > H1760 (Kainong) > H37 (Yuhua) > H71 (Kainong), considering the highest to the lowest total fatty acid and total unsaturated fatty acid contents (Figure 2c). Therefore, these results suggest that OA and linoleic acid were the main components of fatty acids in peanuts, accounting for over 66% of the total fatty acids. The high-OA group, especially H16 and H13 of the Jihua series, had a higher nutritional value than non-high-OA peanuts because of the beneficial functions of OA and richer fatty acid content.

### 3.2. OXITEST Analysis

**Figure 3:**
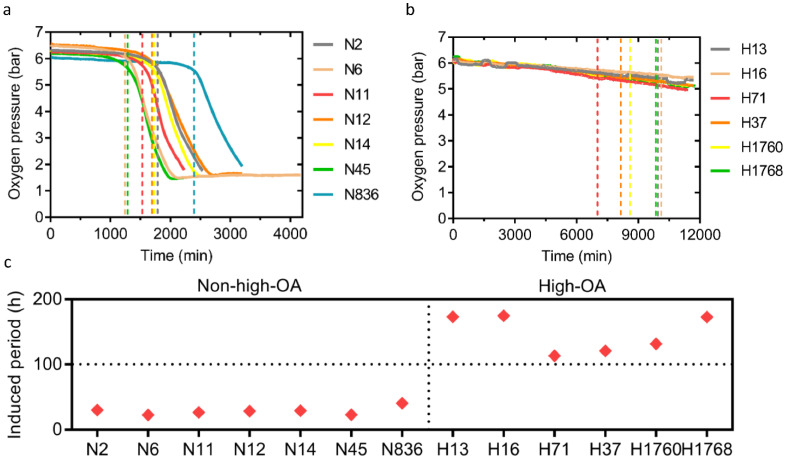
Oxidation stability of 13 peanut cultivars by OXITEST Oxidation Test Reactor. (**a**,**b**) Oxygen consumption curve based on OXITEST data for 15 g non-high-OA peanut cultivars and high-OA peanut cultivars at 90 °C, respectively. OA, oleic acid. (**c**) Scatter plot showing oxidation induced period (IP) of non-high-OA peanut cultivars and high-OA peanut cultivars.

An OXITEST instrument performed the oxidative stability analysis and determined the oxidation curves and IP values of 13 different peanut cultivars (Figure 3a–c). The average IP value of high-OA peanuts was 5.2-fold higher (147.7 ± 29.0 h) than non-high-OA peanuts (28.4 ± 6.0). Within the high-OA group, H16 (Jihua), H13 (Jihua), and H1768 (Kainong) were the top three cultivars with the longest delayed oxidation time (over 170 h). These results suggest that high-OA peanuts had better oxidation stability, indicating that peanuts with high OA had a longer shelf life, a wider application space, and higher economic value. Different high-OA cultivars had different oxidation stability, and the Jihua series cultivars had the most optimal oxidation stability.

### 3.3. Generating Lipid Profiles in Peanuts

The study used a classical method to extract lipids following the METB extraction. The method showed good lipid recovery using five internal standards, including glycerophospholipids, sphingolipids, and glycolipids, with 87.1–98.9% recovery rates [27]. The clustering of quality control samples (QCs) obtained by mixing 20 μL of all individual samples determined the robustness of the analytical procedure. The QCs clustered close to the PCA plot center across the entire sequence, suggesting the high quality of data acquisition (Figure S1).

**Figure 4:**
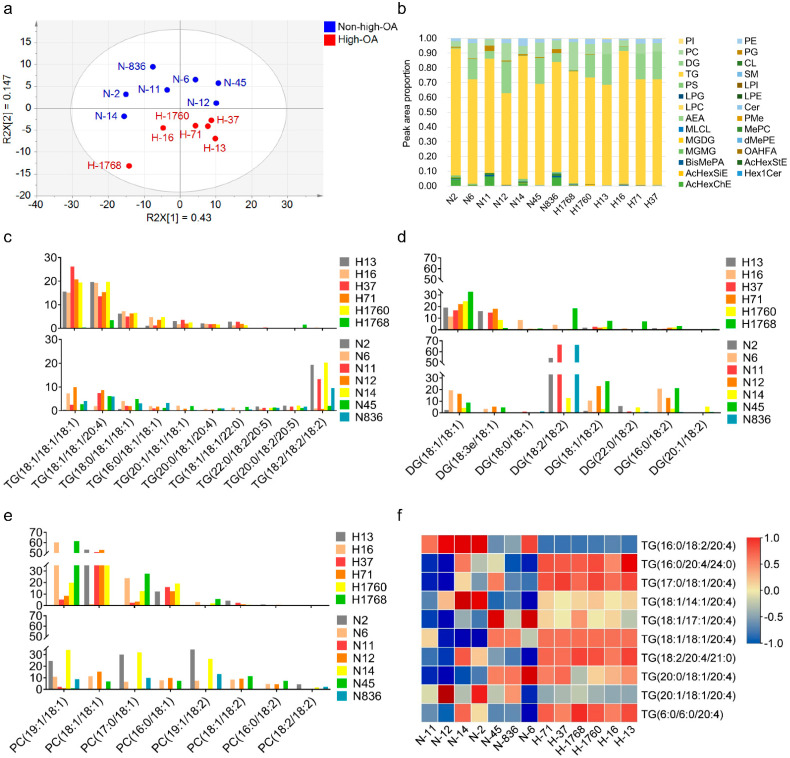
Lipidomics profiles detected in 13 peanut cultivars. (**a**) PCA score plot for 13 peanut cultivars. (**b**) Content of major lipid profiles. (**c**) Distribution of triglyceride profiles. (**d**) Distribution of diacylglycerol profiles. (**e**) Distribution of glycerophospholipids profiles. (**f**) Heatmap showing the content difference in ARA-bonded lipid molecules between high-OA peanut cultivars and non-high-OA peanut cultivars. ARA, arachidonic acid; OA, oleic acid.

**Figure 5:**
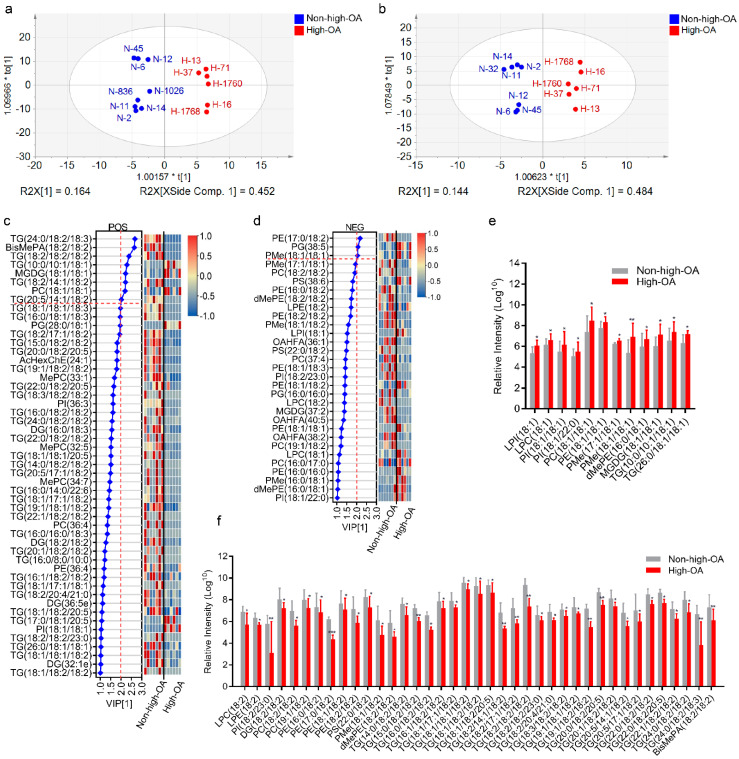
Distribution of identified differently expressed lipids in high-OA peanut cultivars compared to non-high-OA peanut cultivars. (**a**,**b**) PLS-DA score plot of lipid species under positive and negative scan modes, respectively. (**c**,**d**) VIP scores of individual lipids in PLS-DA under positive and negative scan modes, respectively. (**e**) Upregulated lipid species with C18:1 in the profile of high-OA peanut cultivars vs. non-high-OA peanut cultivars. (**f**) Downregulated lipid species with C18:2 in the profile of high-OA peanut cultivars vs. non-high-OA peanut cultivars. The asterisk denotes a significance, * *p* < 0.05, ** *p* < 0.01, *** *p* < 0.001.

In the PCA models, these 13 cultivars clustered into two major groups, following the degree of ploidy, thus, confirming the sample clustering observed in the preliminary set (Figure 4a). The sample data under positive and negative models were reliable since the sum of the goodness of fit X [1] (R2X [1]) and R2X [2] explained over 55% of the total variance. Concurrently, supervised models like OPLS-DA were constructed to highlight the differences between samples. The PLS-DA score plot demonstrated the excellent quality of the models under positive and negative ionization modes because the R2 was over 60% of the total variance (Figure 5ab), thus, validating the models.

The study identified 229 lipid features that simultaneously match the coupled tandem MS library (MS2) (Table S2). Of the 229, 157 lipid features were detected in the positive ionization mode and 72 in the negative ionization mode. The common ion, selected as a representative example, scored 714.5075 (PE 16:1/18:1) on the corresponding spectra (Figure S2). Identifying *m/z* 714.5075 is interpretable from the characteristic fragment ions. The ion *m/z* 281.2483 indicates the 18:1 fatty acid chain, and the ion *m/z* 253.2173 indicates the 16:1 fatty acid chain, fragmented from glyceride bonds at *sn*-1 and *sn*-2 positions. Over 25 lipid families were detected in these peanut cultivars (Figure 4b). The major lipid families in the peanut samples were triglyceride (TG), diglyceride (DG), phosphatidylcholine (PC), and phosphatidylethanolamine (PE). The average TG, DG, and PC content in the peanut samples accounted for 74.74, 10.85, and 7.81% of the total lipids, respectively. The TG content had the greatest effect on total lipid accumulation, accounting for > 70% of the total lipid content.

The study further analyzed the composition of fatty acid species in the TG, DG, and PC profiles. About 200 TGs, 20 DGs, and 20 PCs were synthesized in peanuts and several top representative species with unsaturated fatty acid chains (Figure 4c–e). In the high-OA peanuts, the prominent TG species were TG (18:1/18:1/18:1), TG (18:1/18:1/20:4), and TG (18:0/18:1/18:1). The three TG species were significantly higher (*p* < 0.05) in high-OA peanuts than non-high-OA peanuts. However, the prominent TG species in the non-high-OA were TG (18:2/18:2/18:2), TG (20:0/18:2/20:5), and TG (22:0/18:2/20:5), which were significantly higher (*p* < 0.05) than the high-OA peanuts. Furthermore, DG (18:1/18:1), DG (18:3e/18:1) and PC (18:1/18:1) were the prominent DG and PC species in high-OA peanuts. In the non-high-OA peanuts, the predominant DG and PC profiles were DG (18:2/18:2), DG (16:0/18:2), and PC (19:1/18:2). Additionally, ARA (C20:4) was the most common fatty acid incorporated into the TGs. The TGs heatmap with ARA indicated that a larger proportion of ARA containing lipid molecules accumulated in high-OA peanuts (Figure 4f). The content of TG, DG, and PC with OA and ARA increased in high-OA than non-high-OA peanuts.

### 3.4. Statistical Analyses for High-OA and Non-High-OA Peanut Differentiation

Statistical analyses were performed using the PLS-DA module in SIMCA software and heatmap module in TBtools software. The PLS-DA model classified different types of peanuts based on the available lipids. High-OA and non-high-OA peanuts were classified according to the first two principal components, and the cumulative contribution rates under positive and negative scan modes were 62.8, 61.6%, respectively (Figure 5a,b). A total of 51 and 31 lipid species variables under positive and negative modes significantly contributed to the VIP (>1) (Figure 5c,d). Sixteen of the differently expressed lipids (12 lipids with OA) were upregulated, and 66 (39 lipids with LA) were downregulated in the high-OA than the non-high-OA group.

Further analysis assessed the relative intensity changes of lipid species with carbon atoms C 18:1 and C 18:2. Approximately 75% of the upregulated lipids were C 18:1 bound lipids, and 59% of the downregulated lipids were C 18:2 bound lipids (Figure 5e,f). Furthermore, 11 lipids (VIP > 2) were indicators between the high-OA and the non-high-OA peanut groups under positive and negative ions. Among these, six lipids (TG 18:2/18:2/18:2, TG 24:0/18:2/18:3, TG 20:5/14:1/18:2, TG 18:2/14:1/18:2, PE 17:0/18:2, BisMePA 18:2/18:2) with carbon atoms C 18:2 and a polyunsaturated lipid (PG 38:5) also showed a decrease in content. The other four lipids (PMe 18:1/18:1, PC 18:1/18:1, MGDG 18:1/18:1, TG 10:0/10:1/18:1) were more abundant in high-OA peanuts. Altogether, the content of these lipid molecules exhibited distinct patterns between the two peanut groups. The lipids with C 18:1 were upregulated, while lipids with C 18:2 were downregulated.

Differences in lipid composition between the high-OA peanuts and the non-high-OA peanuts were clarified. The analysis of lipid composition between high-OA peanut cultivars showed seven enriched, representative functional lipid classes in high-OA-peanuts (Figure S3a). The heatmap illustrated that H37 (Yuhua), H71 (Kainong), and H13 (Jihua) had high contents of CL, PS, PI, PC, PE, and DG, and these lipids are important for human health.

TG is a major health risk factor, and consuming higher than normal levels could induce cardiovascular diseases like atherosclerosis [28]. The heatmap displayed that the contents of TGs were relatively low in H1768 (Kainong) but were upregulated in H13 (Jihua), H37 (Yuhua), and H71 (Kainong) (Figure S3b). These results demonstrated the differences in lipid composition among high-OA peanut cultivars. Lipidomics using UPLC-Q-TOF-MS potentially assess the quality and identify different peanut cultivars.

## 4. Discussion

Peanut is an economically important crop rich in oil, and lipid oxidation stability is important for evaluating peanut quality. An OXITEST reactor with two separate oxidation chambers was connected to an oxygen tank and a testing computer with the VELP OXISoft software [29] to measure the peanut oxidation stability. The advantage of this instrument is that it directly measures the oxidation stability of whole foods without requiring sample pretreatment for lipid separation. The device indicates the induction period, rate, and acceleration of the autoxidation process, and the total amount of oxygen consumed by the product. The reactor also performs the freshness test, formula comparison, packaging comparison and estimated shelf life, widely used parameters in the food industry [30]. Therefore, the study used the OXITEST reactor to test lipid oxidation stability.

When the OA content was >60%, and linoleic acid content was <3%, the IP values of the peanut cultivars were over 110 h. However, the IP values of the peanut cultivars were <40 h when the OA content was <30% and linoleic acid content was >40%. Thus, oxidation stability is directly proportional to the OA content and inversely proportional to linoleic acid, consistent with previous studies [31,32]. Besides free fatty acids, the study analyzed the relationship between other lipid classes and oxidation stability. Various substances, including DG, LPC, PC, PS, and TG, were negatively correlated with oxidation stability (Figure S3c). This observation is consistent with reports by Mitchell and Stephanie [33,34], which demonstrated that the substances are susceptible to oxidation because of their high degree of unsaturated content. Lipid composition affects lipid oxidation, nutritional value and other properties [35,36]. Therefore, understanding peanut lipid composition is crucial for improving its application.

The GC–MS identified 16 kinds of free fatty acids. More lipid species were identified than previous studies, likely due to the different analytical methods we used [37]. It is worth noting that the OA content of high-OA peanuts is much higher than the average content of peanuts (43.15%) [38]. Moreover, the study found that the content of OA and LA was significantly different between high-OA and non-high-OA peanut cultivars through fatty acid analysis.

This study used lipidomic methods to compare the quality of 13 peanut cultivars following the main characteristics of the UPLC-Q-TOF-MS within the lipid database. The results identified 27 subclasses of 229 lipids in the 13 peanut cultivars. The lipidomics showed differences in the lipid composition of the high-OA peanut cultivars. Therefore, this study confirmed that lipidomics could be used widely in food analyses, and is potentially a major tool for cultivar identification and food quality analysis. Nevertheless, the study was limited by the small sample size and unoptimized lipidomics method, which can be solved in future studies.

## 5. Conclusions

This study used UPLC-Q-TOF-MS, lipidomics and free fatty acid analysis to determine the lipidomic characteristics and free FAs of 13 various peanut cultivars and compared their lipid composition. Furthermore, OXITEST analysis elucidated the lipid oxidation stability of different peanut cultivars. Therefore, the lipid composition of high-OA peanuts was better than non-high-OA peanuts, especially the OA content. Meanwhile, the oxidation stability of high-OA peanuts was significantly better than the non-high-OA peanuts because of the moderate lipid unsaturation, which means a longer shelf. In addition, the lipid composition of high-OA peanut cultivars varied, and 11 lipid molecules are potential indicators for identifying high-OA and non-high-OA peanut cultivars, respectively. These findings provide the basic knowledge for developing and utilizing different peanut cultivars and reveal the application prospects of lipidomics as a new tool for food quality assessment.

## Supplementary Materials

The following are available online at https://www.mdpi.com/article/10.3390/foods11010004/s1, Figure S1: PCA score plot based on the mass spectrometry data of quality control (QC) under positive and negative scan modes, respectively. Figure S2: MS/MS spectrum and fragmentation pathways of the [M + H] ^+^ at *m/z* 714.5073 of PE (16:1/18:1). Figure S3: (a) Heatmap of multiple functional lipids. CL, Cardiolipin; PS, phosphatidylserine; PG, phosphatidylglycerol; PI, phosphatidylinositol; PC, phosphatidylcholine; PE, phosphatidylethanolamine; DG, diglyceride. (b) Heatmap of triglyceride (TG). (c) Heatmap of consistency analysis of lipid and oxidation stability. IP, the induction periods. The asterisk denotes a significance, * *p* < 0.05, ** *p* < 0.01, *** *p* < 0.001. Table S1: Fatty acid contents in different peanut cultivars. Table S2: Lipid composition of different peanut cultivars.
