# Supplementary material for: A Comparison of Lipid Contents in Different Types of Peanut Cultivars Using UPLC-Q-TOF-MS-Based Lipidomic Study
Source: Foods. 2021 Dec 21;11(1):4. doi: 10.3390/foods11010004 (PMC8750182; doi:10.3390/foods11010004)
Supplement: Supplementary file 1 [file foods-11-00004-s001.zip › Table S1.pdf]

Table S1: Fatty acid contents in different peanut cultivars (mg · g^{-1} W).

The number	Name	CAS #	Mol.Form	Jihua16-1	Jihua16-2	Jihua16-3	Jihua13-1	Jihua13-2	Jihua13-3	Kainong17	Kainong17	Kainong17	Kainong17	Kainong17	Kainong17
1	Hexadecer	1120 - 25 -	C17H32O2	0.43	0.45	0.46	0.52	0.49	0.51	0.35	0.36	0.36	0.35	0.39	0.37
2	Hexadecar	112 - 39 - (	C17H34O2	23.04	22.56	23.28	22.18	21.37	21.96	19.96	20.22	20.97	18.66	19.83	18.85
3	Cyclopropa	10152 - 61	C18H34O2	0.16	0.15	0.15	0.16	0.16	0.14	0.10	0.10	0.11	0.13	0.12	0.10
4	Heptadeca	1731 - 92 -	C18H36O2	0.25	0.23	0.24	0.19	0.21	0.21	0.18	0.16	0.20	0.21	0.20	0.20
5	9,12-Octac	112 - 63 - (	C19H34O2	2.53	3.03	2.14	2.06	2.09	2.92	2.34	2.71	2.55	1.86	2.17	2.18
6	9-Octadec	1937 - 62 -	C19H36O2	145.25	146.73	148.65	137.36	138.07	136.29	134.78	133.96	138.57	121.80	125.01	121.28
7	Methyl ste	112 - 61 - 8	C19H38O2	22.27	22.39	23.02	15.87	14.98	15.71	16.28	16.67	17.50	15.39	16.11	14.33
8	10-Nonade	56599 - 83	C20H38O2	0.07	0.08	0.07	0.05	0.05	0.04	0.05	0.04	0.03	0.05	0.06	0.06
9	cis-Methyl	2390 - 9 - 2	C21H40O2	4.83	4.83	4.91	4.82	4.72	4.72	4.20	4.26	4.36	3.94	4.20	4.02
10	Eicosanoic	1120 - 28 -	C21H42O2	8.79	8.83	8.96	6.36	6.18	6.34	6.69	6.74	6.97	6.29	6.64	5.99
11	Heneicosai	6064 - 90 -	C22H44O2	0.08	0.07	0.07	0.05	0.05	0.05	0.06	0.04	0.06	0.04	0.05	0.04
12	13-Docose	1120 - 34 -	C23H44O2	0.29	0.26	0.34	0.31	0.30	0.27	0.24	0.25	0.24	0.21	0.24	0.23
13	Docosanoi	929 - 77 - 1	C23H46O2	11.69	11.52	11.93	9.46	9.14	9.18	9.89	9.78	10.21	8.51	9.04	8.32
14	Tricosanoi	2433 - 97 -	C24H48O2	0.11	0.12	0.11	0.11	0.10	0.10	0.10	0.09	0.08	0.08	0.08	0.08
15	Tetracosar	5129 - 66 -	C16H32O2	5.26	5.19	5.42	5.06	4.84	4.94	4.32	4.38	4.46	4.48	4.74	4.45
16	Hexacosan	5802 - 82 -	C27H54O2	0.70	0.70	0.72	0.71	0.66	0.67	0.53	0.47	0.50	0.67	0.67	0.67
			SUM	225.75	227.15	230.48	205.28	203.41	204.05	200.07	200.24	207.15	182.66	189.56	181.19
			Oleic acid conte	64.34	64.60	64.50	66.91	67.88	66.79	67.37	66.90	66.89	66.68	65.95	66.93
			Linoleic acid cor	1.12	1.33	0.93	1.00	1.03	1.43	1.17	1.35	1.23	1.02	1.14	1.20

The number	Name	Yuhua37-1	Yuhua37-2	Yuhua37-3	Kainong71	Kainong71	Kainong71	Zhanjiang2	Zhanjiang2	Zhanjiang2	Zhonghua6	Zhonghua6	Zhonghua6	Heyou11-1	Heyou11-2	Heyou11-3
1	Hexadecer	0.36	0.34	0.33	0.29	0.32	0.28	0.18	0.22	0.18	0.10	0.09	0.09	0.09	0.10	0.10
2	Hexadecar	17.48	17.95	17.64	15.99	16.76	16.14	23.44	25.10	24.00	10.61	10.14	9.51	14.12	14.60	14.73
3	Cyclopropa	0.10	0.10	0.10	0.10	0.10	0.10	0.05	0.08	0.06	0.06	0.06	0.05	0.04	0.03	0.03
4	Heptadeca	0.16	0.15	0.16	0.15	0.14	0.17	0.17	0.21	0.18	0.13	0.11	0.11	0.11	0.12	0.12
5	9,12-Octac	3.39	3.01	3.47	2.32	2.98	2.17	72.68	78.26	71.43	29.38	26.63	25.39	40.41	42.65	44.59
6	9-Octadec	113.69	112.58	113.21	101.89	104.44	106.35	43.00	45.10	43.29	19.97	19.04	18.57	27.04	27.27	27.68
7	Methyl ste	13.22	13.75	13.23	11.37	11.98	12.32	10.90	12.40	10.57	3.95	3.79	3.54	6.97	7.28	7.48
8	10-Nonade	0.04	0.05	0.05	0.05	0.05	0.03	0.03	0.03	0.03	0.02	0.03	0.02	0.01	0.02	0.02
9	cis-Methyl	3.75	3.78	3.82	3.50	3.67	3.48	2.00	2.29	1.89	0.84	0.80	0.72	1.03	1.12	1.10
10	Eicosanoic	5.44	5.47	5.53	4.83	4.86	4.83	4.57	5.29	4.48	1.81	1.72	1.60	2.53	2.65	2.64
11	Heneicosai	0.05	0.04	0.03	0.04	0.04	0.03	0.05	0.05	0.04	0.02	0.02	0.01	0.02	0.03	0.02
12	13-Docose	0.24	0.23	0.24	0.21	0.22	0.20	0.10	0.11	0.08	0.03	0.03	0.03	0.04	0.05	0.05
13	Docosanoi	8.00	8.10	8.02	7.02	7.21	7.05	7.17	8.02	7.12	3.05	2.91	2.71	3.83	3.99	3.94
14	Tricosanoi	0.08	0.08	0.07	0.07	0.08	0.07	0.07	0.10	0.09	0.04	0.04	0.03	0.04	0.03	0.04
15	Tetracosar	4.17	4.16	4.19	3.73	3.88	3.68	3.25	3.61	3.02	1.32	1.27	1.14	1.65	1.73	1.68
16	Hexacosan	0.56	0.59	0.58	0.51	0.44	0.46	0.40	0.47	0.35	0.15	0.16	0.13	0.15	0.18	0.18
	SUM	170.72	170.38	170.66	152.06	157.17	157.37	168.06	181.35	166.80	71.50	66.83	63.67	98.08	101.87	104.39
	Oleic acid conte	66.59	66.07	66.33	67.01	66.45	67.58	25.58	24.87	25.95	27.93	28.49	29.17	27.57	26.77	26.51
	Linoleic acid cor	1.98	1.77	2.03	1.52	1.90	1.38	43.25	43.15	42.82	41.09	39.84	39.88	41.20	41.86	42.71

The number	Name	Heyou12-1	Heyou12-2	Heyou12-3	Fuhua14-1	Fuhua14-2	Fuhua14-3	Yueyou45-	Yueyou45-	Yueyou45-	Guihua836	Guihua836	Guihua836-3
1	Hexadecer	0.16	0.15	0.14	0.22	0.19	0.19	0.08	0.09	0.08	0.13	0.21	0.15
2	Hexadecar	19.64	18.57	18.68	27.18	25.90	25.74	12.52	12.57	12.74	28.70	28.26	29.54
3	Cyclopropa	0.07	0.06	0.05	0.08	0.06	0.08	0.04	0.04	0.03	0.07	0.01	0.09
4	Heptadeca	0.18	0.17	0.15	0.22	0.19	0.20	0.12	0.11	0.11	0.18	0.31	0.23
5	9,12-Octac	54.09	51.18	50.59	74.59	71.67	71.36	36.58	36.22	37.27	78.64	77.30	78.83
6	9-Octadec	41.22	40.95	41.92	40.99	40.16	42.01	20.18	19.78	19.92	43.04	43.84	45.29
7	Methyl ste	9.30	8.59	8.93	10.47	9.97	9.89	6.04	6.00	6.06	7.10	8.45	8.06
8	10-Nonade	0.04	0.03	0.03	0.03	0.03	0.03	0.02	0.01	0.02	0.03	0.04	0.04
9	cis-Methyl	1.84	1.64	1.71	2.05	1.94	1.94	1.01	0.98	0.99	2.32	3.41	2.65
10	Eicosanoic	3.79	3.43	3.58	4.07	3.70	3.67	2.33	2.29	2.36	3.01	4.50	3.54
11	Heneicosai	0.04	0.03	0.03	0.05	0.04	0.05	0.03	0.03	0.03	0.03	0.05	0.04
12	13-Docose	0.09	0.08	0.08	0.12	0.10	0.11	0.05	0.05	0.05	0.13	0.17	0.14
13	Docosanoi	5.86	5.45	5.53	6.43	6.06	5.89	4.00	3.86	3.92	6.01	7.77	6.81
14	Tricosanoi	0.06	0.06	0.06	0.09	0.07	0.09	0.05	0.04	0.03	0.06	0.13	0.08
15	Tetracosar	2.66	2.43	2.43	3.09	2.85	2.87	1.40	1.41	1.40	3.00	4.35	3.38
16	Hexacosan	0.29	0.24	0.26	0.30	0.30	0.24	0.14	0.13	0.13	0.28	0.40	0.31
	SUM	139.32	133.07	134.18	169.99	163.24	164.35	84.57	83.60	85.14	172.74	179.19	179.19
	Oleic acid conte	29.59	30.77	31.24	24.12	24.60	25.56	23.86	23.66	23.40	24.92	24.47	25.28
	Linoleic acid cor	38.82	38.46	37.70	43.88	43.91	43.42	43.25	43.33	43.77	45.52	43.14	43.99
