# Supplementary material for: A Comparison of Lipid Contents in Different Types of Peanut Cultivars Using UPLC-Q-TOF-MS-Based Lipidomic Study
Source: Foods. 2021 Dec 21;11(1):4. doi: 10.3390/foods11010004 (PMC8750182; doi:10.3390/foods11010004)
Supplement: Supplementary file 1 [file foods-11-00004-s001.zip › Table S2.pdf]

Table S2: Lipid composition of different peanut cultivars.

Ion	LipidIon	LipidGroup	Class	FattyAcid	FA1	FA2	FA3	ObsMz	IonFormula	Area[Heyou	Area[Heyou	Area[Fuhua	Area[Zhanji	Area[Yueyo	Area[Guihu	Area[Zhong	Area[Kainor	Area[Yuhua	Area[Kainor	Area[Kainor	Area[Jihua1	Area[Jihua13]
NEG	Cer(d34:2)	Cer(d34:2+O)-H	Cer	(d34:2+O)	(d34:2+O)			550.484083	C34 H64 O4 N1	619792.36	1818608.5	30494.795	14375.11	243367.55	10341.19	45641.838	76228.382	66159.797	28368.156	106268.66	19002.681	7256704.2
NEG	Cer(t17:1/25:0+O)	Cer(t42:1+O)-H	Cer	(t17:1_25:0+O)	(t17:1)	(25:0+O)		680.619848	C42 H82 O5 N1	155739.08	95428.33	10958483	4042045.4	2507316	59605118	7872129.1	4849076.5	5850667.6	9794797.6	16687064	18339677	112892.95
NEG	Cer(t32:0)	Cer(t32:0)+HCOO	Cer	(t32:0)	(t32:0)			572.489563	C33 H66 O6 N1	101063.28	1251896.7	203042.14	1.055	1387630.4	8279.2371	928877.61	1977239.8	1176737.8	0.64	1748922.9	1994270.5	30920.945
NEG	Cer(t34:0)	Cer(t34:0)+HCOO	Cer	(t34:0)	(t34:0)			600.520863	C35 H70 O6 N1	0	4209.2232	9534.0888	100047.5	18047.115	3725785.5	21796.991	8242.0884	5120.269	7642.6728	62993.355	75143.052	0
NEG	Cer(t42:1+O)	Cer(t42:1+O)+HCOO	Cer	(t17:1_25:0+O)	(t17:1)	(25:0+O)		726.625328	C43 H84 O7 N1	11.432	230001.72	5629741.4	4899166.7	45950.416	24985996	4600646.8	2737154.5	2801227.3	7104161.4	8435784.6	9264611.2	41546.115
NEG	CL(74:10)	CL(74:10)-2H	CL	(74:10)	(74:10)			735.478839	C83 H140 O17 P2	1160562.5	1282924.3	73588.978	45917.338	1919296.6	3292.6137	2725.4556	2760649.2	4080595.3	30193.995	1055701.7	5695.9809	2744500.8
NEG	CL(87:9)	CL(87:9)-2H	CL	(87:9)	(87:9)			827.588389	C96 H168 O17 P2	1601596.4	2516749.8	14577808	9903697.6	1673379.2	10851374	21229857	12966159	8139343.2	33131311	12778575	20418163	287660.89
NEG	dMePE(16:0/18:1)	dMePE(34:1)-H	dMePE	(16:0_18:1)	(16:0)	(18:1)		744.554881	C41 H79 O8 N1 P1	16559014	14968312	61705.127	44836.397	21054458	1100293.5	37232.974	13595640	17012885	139258.15	13940710	1934510.7	18527637
NEG	dMePE(18:2/18:2)	dMePE(36:4)-H	dMePE	(18:2_18:2)	(18:2)	(18:2)		766.539231	C43 H77 O8 N1 P1	20143.964	60740.1	2586454	8212958.4	88069.081	11484173	3840023.4	139072.6	91279.895	46015.897	46489.29	10907.329	10641.181
NEG	Hex1Cer(t34:2)	Hex1Cer(t34:2)+HCOO	Hex1Cer	(t34:2)	(t34:2)			758.542388	C41 H76 O11 N1	1944.5211	5356.5792	24199326	11899355	906842.34	32539459	20644132	4485276.2	104217.55	27060312	6234433.7	15708405	243049.08
NEG	Hex2Cer(d33:2)	Hex2Cer(d33:2)-H	Hex2Cer	(d33:2)	(d33:2)			844.579168	C45 H82 O13 N1	86207.471	23213.94	7699.7037	9517.9617	29477.088	14909.719	13819.26	1398.648	283.2024	13522.45	22415.951	16467.971	5918849.4
NEG	LPC(16:0)	LPC(16:0)+HCOO	LPC	(16:0)	(16:0)			540.330696	C25 H51 O9 N1 P1	1183625.1	1227725.8	23977.492	69810.918	855436.32	6820.8648	137352.73	446955.29	1034423	30168.631	349158.22	40348.808	1715173.6
NEG	LPC(18:1)	LPC(18:1)+HCOO	LPC	(18:1)	(18:1)			566.346346	C27 H53 O9 N1 P1	4865920.3	3878956.9	190836.92	656954.19	4808847.2	362739.39	2299993.6	9232132.9	11671774	1032185.7	4305888.2	584501.03	14388784
NEG	LPE(16:0)	LPE(16:0)-H	LPE	(16:0)	(16:0)			452.278266	C21 H43 O7 N1 P1	14349.864	42972.508	1392482.9	4743149	18537.877	4700057.9	3020588.9	829784.57	19703.997	2018525.5	1257685.9	898459.25	5588.726
NEG	LPE(18:1)	LPE(18:1)-H	LPE	(18:1)	(18:1)			478.293916	C23 H45 O7 N1 P1	25684.119	3643.6332	4496974.3	7484469.5	15401.657	8172692.1	7466692.6	4135176	2041140.1	13073229	5992089.8	4472685.4	28926.713
NEG	LPE(18:2)	LPE(18:2)-H	LPE	(18:2)	(18:2)			476.278266	C23 H43 O7 N1 P1	355575.66	172953.99	1400899.7	5753388.1	276106.82	6027576.4	2443698.4	418386.69	401708.03	260131.99	111313.85	27657.817	264896.66
NEG	LPG(16:0)	LPG(16:0)-H	LPG	(16:0)	(16:0)			483.272847	C22 H44 O9 N0 P1	0	0	119408.03	520252.56	0	474582.22	3555.915	3298.7556	1646.286	934291.96	2116.833	26041.041	0
NEG	LPI(16:0)	LPI(16:0)-H	LPI	(16:0)	(16:0)			571.288892	C25 H48 O12 N0 P1	572034.7	620102.59	2605.068	121722.65	587770.2	127255.51	10265.174	521781.85	686178.73	70503.653	130439.11	3551.526	1331459.9
NEG	LPI(18:1)	LPI(18:1)-H	LPI	(18:1)	(18:1)			597.304542	C27 H50 O12 N0 P1	861998.49	610017.02	24751.836	202788.02	921926.77	163923.79	38008.032	2392143.6	2459355.1	388299.72	1121008.3	152527.76	4088069.8
NEG	MGDG(16:0/18:1)	MGDG(34:1)+HCOO	MGDG	(16:0_18:1)	(16:0)	(18:1)		801.573354	C44 H81 O12	70293.134	705044.48	5261389.1	5321092.7	2665085.9	13371307	14891845	4372594.2	2703222.3	9469840.8	7546569.9	3334865.5	76669.246
NEG	MGDG(18:1/18:1)	MGDG(36:2)+HCOO	MGDG	(18:1_18:1)	(18:1)	(18:1)		827.589004	C46 H83 O12	26920.385	175573.77	5667475	56852.184	1192159.1	1898560	8947137.4	3096941.1	11586406	5182252.8	20789160	15150524	37370.34
NEG	MGDG(18:1/18:2)	MGDG(36:3)+HCOO	MGDG	(18:1_18:2)	(18:1)	(18:2)		825.573354	C46 H81 O12	14993.329	75656.631	11087807	11932715	59551.208	17748776	14005911	684012.86	550358.5	2498977.2	1062201.5	914281.32	19757.942
NEG	MGDG(18:2/18:2)	MGDG(36:4)+HCOO	MGDG	(18:2_18:2)	(18:2)	(18:2)		823.557704	C46 H79 O12	9876.2166	16338.189	947262.57	988164.9	146053.61	896796.62	2632564.7	283588.36	1514940.2	139077.42	515113.73	50442.26	29107.179
NEG	MGDG(37:2)	MGDG(37:2)+HCOO	MGDG	(37:2)	(37:2)			841.604654	C47 H85 O12	25689.425	1049562.5	2319247.3	17876.245	3987576.9	50570.454	5064811.8	3748869.7	15550918	449432.76	17885439	9543652.2	1389220.8
NEG	MGDG(37:3)	MGDG(37:3)+HCOO	MGDG	(37:3)	(37:3)			839.589004	C47 H83 O12	36689.211	91217.736	3450001.1	1223882.1	100399.14	2276105.6	6982237.5	36887.637	165049.57	100244.48	3351151	2757065.5	58908.441
NEG	MGMG(32:4)	MGMG(32:4)+HCOO	MGMG	(32:4)	(32:4)			753.515839	C42 H73 O11	94629.141	149626.61	3399294.4	5248643.5	681909.23	8619029.8	4745619.6	111240.87	15575.882	3996680	2433182.6	2405556.1	45032.417
NEG	MLCL(52:4)	MLCL(52:4)-2H	MLCL	(52:4)	(52:4)			580.364007	C61 H110 O16 P2	675975.88	121082	5358555.2	13133598	129482.4	13301404	15316945	23317473	23848863	8359651.2	14131768	5943744.6	62129.924
NEG	OAHFA(36:1)	OAHFA(36:1)-H	OAHFA	(36:1)	(36:1)			563.504484	C36 H67 O4	676806.45	842127.11	29152.163	0	2109878.3	36373.999	1791834.9	19052695	12382434	0	2818946	1934748.5	7222533.2
NEG	OAHFA(38:2)	OAHFA(38:2)-H	OAHFA	(38:2)	(38:2)			589.520134	C38 H69 O4	544940.44	903130.99	0	0	712530.63	0	188619.55	1566308.5	960352.23	0	526897.7	258028.11	933111.8
NEG	OAHFA(38:3)	OAHFA(38:3)-H	OAHFA	(38:3)	(38:3)			587.504484	C38 H67 O4	69512.633	44890.364	88967.052	1821302.8	101113.04	1048076.4	594885.83	45310.684	597235.35	1436783.8	10456.889	27875.821	637128.8
NEG	OAHFA(38:4)	OAHFA(38:4)-H	OAHFA	(38:4)	(38:4)			585.488834	C38 H65 O4	1335.7251	22218.032	7355453.2	7392763.2	3261.9039	4529084.8	20401514	17549433	3514707.1	17205943	18727746	55285796	25906.848
NEG	OAHFA(40:4)	OAHFA(40:4)-H	OAHFA	(40:4)	(40:4)			613.520134	C40 H69 O4	2432749.4	2828496.9	8162.0304	0	3889079.4	12999.887	5845.1196	7395024.3	9166276	1.585	4410501.6	2006339	7064642.6
NEG	OAHFA(40:5)	OAHFA(40:5)-H	OAHFA	(40:5)	(40:5)			611.504484	C40 H67 O4	3077.129	27626.064	1139028.2	2008471.8	15582.948	2033749	1489819.8	7257.6357	25088.487	111936.72	4323.0348	165611.55	10868.876
NEG	PC(15:0/18:1)	PC(33:1)+HCOO	PC	(15:0_18:1)	(15:0)	(18:1)		790.560361	C42 H81 O10 N1 P1	935514.96	1716902.1	6140.8074	0	2436800.2	63776.664	258131.13	1816862.1	624580.29	4908.456	452736.99	21982.641	1000899.6
NEG	PC(16:0/17:0)	PC(33:0)+HCOO	PC	(16:0_17:0)	(16:0)	(17:0)		792.576011	C42 H83 O10 N1 P1	69496.962	71643.356	10792151	18789666	1588172.8	20334419	9898661.5	4497522.4	31388.616	6663826.6	487703.95	780200.68	10804.709
NEG	PC(17:0/18:1)	PC(35:1)+HCOO	PC	(17:0_18:1)	(17:0)	(18:1)		818.591661	C44 H85 O10 N1 P1	7040508.5	27063325	449130158	445783891	53277168	455523635	507838924	161544990	110901785	533627960	374043164	256969307	2831821.4
NEG	PC(18:2/18:2)	PC(36:4)+HCOO	PC	(18:2_18:2)	(18:2)	(18:2)		826.560361	C45 H81 O10 N1 P1	233663.85	2975858	22426961	63931984	1569281.7	97476329	30251902	1493392.2	1506142.4	383796.24	251109.58	190676.57	86490.194
NEG	PC(19:0/18:1)	PC(37:1)+HCOO	PC	(19:0_18:1)	(19:0)	(18:1)		846.622961	C46 H89 O10 N1 P1	52972420	51459315	90461.56	266107.43	43020197	3999172	97669.811	40410521	29306342	186156.81	23265479	2933021.3	67893951
NEG	PC(19:1/18:1)	PC(37:2)+HCOO	PC	(19:1_18:1)	(19:1)	(18:1)		844.607311	C46 H87 O10 N1 P1	57353643	92235568	479187550	364403412	71124334	407265358	832059289	394574443	237912420	1.183E+09	587740554	648327097	25509309
NEG	PC(19:1/18:2)	PC(37:3)+HCOO	PC	(19:1_18:2)	(19:1)	(18:2)		842.591661	C46 H85 O10 N1 P1	7365720.2	3600401.2	372631246	511285730	36885583	610322755	566851711	21558759	15029566	110107985	60798902	32842388	390890.16
NEG	PC(36:5)	PC(36:5)+HCOO	PC	(36:5)	(36:5)			824.544711	C45 H79 O10 N1 P1	2059733.8	2348020.9	24859842	8640296.8	712344.06	14189407	22849283	21047646	8476060.6	53760257	22765614	35024661	81731.08
NEG	PC(37:4)	PC(37:4)+HCOO	PC	(37:4)	(37:4)			840.576011	C46 H83 O10 N1 P1	60354307	350562386	117624.8	267360.74	137269894	5321983.9	10958316	553811.64	1615054.3	2088.114	856330.57	178064.69	12362213
NEG	PE(16:0/16:0)	PE(32:0)-H	PE	(16:0_16:0)	(16:0)	(16:0)		690.507931	C37 H73 O8 N1 P1	31304.528	44246.146	3238835	3077187.3	362081.48	4302690.4	3512317.1	1484166.3	34766.928	1065224.6	79129.514	636159.67	0
NEG	PE(16:1/18:1)	PE(16:1/18:1)-H	PE	(16:1)	(18:1)			714.507314	C39 H74 O8 N1 P1	67341773	173014919	142622.67	47453.022	75211024	799707.12	128434.26	21495.471	1184337.5	0.142	194500.1	1.044	3811042.5
NEG	PE(17:0/18:2)	PE(35:2)-H	PE	(17:0_18:2)	(17:0)	(18:2)		728.523581	C40 H75 O8 N1 P1	0	0	978150.03	2683152.6	0	2130138.5	1179191.4	0	0	60660.727	18990.569	0	10041.05
NEG	PE(18:1/18:3)	PE(36:4)-H	PE	(18:1_18:3)	(18:1)	(18:3)		738.517494	C41 H73 O8 N1 P1	67341773	173014919	142622.67	47453.022	75211024	799707.12	128434.26	21495.471	1184337.5	0.142	194500.1	1.044	3811042.5
NEG	PE(18:2/18:2)	PE(36:4)-H	PE	(18:2_18:2)	(18:2)	(18:2)		738.507931	C41 H73 O8 N1 P1	405401.95	691969.44	59566520	154621506	3531383.6	175430281	58260507	2252814.9	1477370.4	1337847.1	1102665.4	877989.59	44954.87
NEG	PG(16:0/16:0)	PG(32:0)-H	PG	(16:0_16:0)	(16:0)	(16:0)		721.502512	C38 H74 O10 N0 P1	4508.9304	6635.9163	2090775.2	4102713.4	12613.053	7352851.8	5164052.9	1.067	4090.6944	177912.49	28780.338	543757.42	0
NEG	PG(16:0/18:1)	PG(34:1)-H	PG	(16:0_18:1)	(16:0)	(18:1)		747.518162	C40 H76 O10 N0 P1	94545.734	3769969	3002.9682	54283.264	8275989.6	62479.206	1714536.3	18194726	11184197	130269.62	7718886.9	1577477.7	29055.296
NEG	PG(38:5)	PG(38:5)-H	PG	(38:5)	(38:5)			795.518162	C44 H76 O10 N0 P1	195903.85	88091.669	0	0	25072.307	0	79310.21	3919681.2	3756278.4	0	879078	117971.18	296872.77
NEG	PI(16:0/18:1)	PI(34:1)-H	PI	(16:0_18:1)	(16:0)	(18:1)		835.534207	C43 H80 O13 N0 P1	109959410	42324863	8564.988	17004.625	31223423	33196.66	28358.101	840952.32	7172864.3	24285.536	46059.168	7029.3375	275594492
NEG	PI(16:0/18:2)	PI(34:2)-H	PI	(16:0_18:2)	(16:0)	(18:2)		833.518557	C43 H78 O13 N0 P1	3647630.7	28724392	11227.232	24576.816	58944979	53845.419	8478.7104	6670155.5	10392082	6419.425	5126870.1	44192.506	148987.93
NEG	PI(16:0/18:3)	PI(34:3)-H	PI	(16:0_18:3)	(16:0)	(18:3)		831.502907	C43 H76 O13 N0 P1	36220.862	5702.7897	0	2818.4436	74798.742	13008.083	22418.497	99306.668	59525.251	0	4385.421	0	982338.81
NEG	PI(17:0/18:2)	PI(35:2)-H	PI	(17:0_18:2)	(17:0)	(18:2)		847.534207	C44 H80 O13 N0 P1	265002.25	30679.44	357782.37	986774.52	284553.2	1721792.7	60860.533	1224110.1	2150366.7	10783.256	573169.05	18243.303	2687600.8
NEG	PI(18:0/18:1)	PI(36:1)-H	PI	(18:0_18:1)	(18:0)	(18:1)		863.565507	C45 H84 O13 N0 P1	35075.161	137752.92	6279291.8	12681034	23857.583	27021673	20472506	3496366.6	883443.47	7793694.6	9182411.6	10576520	89248.816
NEG	PI(18:1/18:2)	PI(36:3)-H	PI	(18:1_18:2)	(18:1)	(18:2)		859.534207	C45 H80 O13 N0 P1	148034.63	8582966.4	0	5848.5633	14779885	125378.29	2311030	5083182.4	9427943.7	4291.7112	3712093.6	142044.29	18907.725
NEG	PI(18:1/22:0)	PI(40:1)-H	PI	(18:1_22:0)	(18:1)	(22:0)		919.628107	C49 H92 O13 N0 P1	141863.65	554578.94	0	0	479006.07	26116.492	18004.53	2019486	2697638.7	30626.158	1276416.4	189462.39	20356.56
NEG	PI(18:1/24:0)	PI(42:1)-H	PI	(18:1_24:0)	(18:1)	(24:0)		947.659407	C51 H96 O13 N0 P1	59188.076	1428295.7	30116.645	0	845414.56	251947.69	459563	7108712.4	8641354.3	97740.909	4212412.6	16496.711	110081.77
NEG	PI(18:2/23:0)	PI(41:2)-H	PI	(18:2_23:0)	(18:2)	(23:0)		931.628107	C50 H92 O13 N0 P1	0	26267.623	747791.79	752013.1	0	3384863.7	1292349.4	0.063	39770.611	10950.779	96996.576	0	0
NEG	PI(32:2e)	PI(32:2e)-H	PI	(32:2e)	(32:2e)			791.507992	C41 H76 O12 N0 P1	13562.093	1117263.9	0	3830.22	2110839.7	15724.296	8819.8284	3304263.3	4611834.5	0	3071875	4116.694	81967.446
NEG	PI(33:1)	PI(33:1)-H	PI	(33:1)	(33:1)			821.518557	C42 H78 O13 N0 P1	61691.855	967607.99	145340.61	934130.01	1972098	648225.14	394096.96	3098802.4	3507792.9	1484597.2	1030544.7	17985.944	118378.94
NEG	PMe(16:0/18:1)	PMe(34:1)-H	PMe	(16:0_18:1)	(16:0)	(18:1)		687.497032	C38 H72 O8 N0 P1	274375.7	29169.481	19094351	16047322	54694.145	21537818	28853132	2668701.6	128225.16	18429.262	2757528.7	4584640	6038.4543
NEG	PMe(17:1/18:1)	PMe(35:2)-H	PMe	(17:1_18:1)	(17:1)	(18:1)		699.497032	C39 H72 O8 N0 P1	2072040.4	1588961.1	1218839.1	2088228.3	2295319.6	1400949.8	1395275.8	4018538	4852676.9	3381254.3	2658753.2	1387650.3	5480768.9
NEG	PMe(18:0/18:1)	PMe(36:1)-H	PMe	(18:0_18:1)	(18:0)	(18:1)		715.528332	C40 H76 O8 N0 P1	86702.067	29080.263	2113903.1	2058148.5	9887.7207	2389238.8	3519624.6	448465.21	28355.674	259578.51	8933.259	787777.26	81557.664
NEG	PMe(18:1/18:1)	PMe(36:2)-H	PMe	(18:1_18:1)	(18:1)	(18:1)		713.512682	C40 H74 O8 N0 P1	24761.066	5726210.1	8699.3958	23510.989	4011381.2	55256.325	4736689.5	90863638	105183235	30484.784	23621732	5116790.8	9185159.3
NEG	PMe(18:1/18:2)	PMe(36:3)-H	PMe	(18:1_18:2)	(18:1)	(18:2)		711.497032	C40 H72 O8 N0 P1	84344.216	56052.686	14992122	30594368	34114.072	18292276	3944590.2	18295.019	49033.646	2269706.7	34984.984	12625.251	42364.752
NEG	PS(11:0/18:1)	PS(29:1)-H	PS	(11:0_18:1)	(11:0)	(18:1)		690.435161	C35 H65 O10 N1 P1	998378.18	614363.22	96073.392	21239.404	3107326.5	16672.117	720982.74	5631849.9	20862923	52211.423	5176467.9	2012820	64625.207
NEG	PS(22:0/18:2)	PS(40:2)-H	PS	(22:0_18:2)	(22:0)	(18:2)		842.591661	C46 H85 O10 N1 P1	10356740	38370787	396515925	546420763	111407190	615422056	601481726	72012909	37271682	43397469	67481247	35438534	143502.57
NEG	PS(36:3)	PS(36:3)-H	PS	(36:3)	(36:3)			784.513411	C42 H75 O10 N1 P1	71608.437	56686.484	27422877	19398414	3257451.9	23776262	25617391	10458524	5843342.9	11312652	17119440	17812053	31562.462
NEG	PS(38:4)	PS(38:4)-H	PS	(38:4)	(38:4)			810.529061	C44 H77 O10 N1 P1	699871.11	29762.386	11770928	3003213.8	3827543.7	4380545.9	13364736	27627578	14695071	6483549	23845426	20312485	114405.32
NEG	PS(38:6)	PS(38:6)-H	PS	(38:6)	(38:6)			806.497761	C44 H73 O10 N1 P1	15349.876	40478.251	2539203.8	6698079.7	40850.015	7592095.4	2969220.6	71097.764	36410.72	8034.332	9695.071	0.093	0.111
NEG	SM(d36:5)	SM(d36:5)+HCOO	SM	(d36:5)	(d36:5)			767.534480	C42 H76 O8 N2 P1	3448790.4	4694307.3	7048.8864	25086.766	4300722.2	13117.686	5428.266	2482278.6	3164433.3	17426.981	788341.39	17652.653	2751122
POS	AcHexChE(24:1)	AcHexChE(24:1)+NH4	AcHexChE	(24:1)	(24:1)			914.780730	C57 H104 O7 N1	4.041E+09	124425338	518949102	1.886E+09	29016161	4.948E+09	169855373	7932799.6	8886269.1	25845446	15761292	34513481	266855.84
POS	AcHexSiE(18:1)	AcHexSiE(18:1)+NH4	AcHexSiE	(18:1)	(18:1)			858.718130	C53 H96 O7 N1	21305085	3191917.9	3024324.6	511835.26	72520.068	17072392	803572.94	18995736	14150243	403701.47	49498075	98023782	371835.35
POS	AcHexStE(18:1)	AcHexStE(18:1)+NH4	AcHexStE	(18:1)	(18:1)			856.702480	C53 H94 O7 N1	1980035.6	28153384	282033.67	519888.82	43366150	816802.18	31259198	3895040.5	4886439	169095.95	3560350.1	156972.19	17225211
POS	AEA(15:1)	AEA(15:1)+NH4	AEA	(15:1)	(15:1)			301.284954	C17 H37 O2 N2	0	1819631.2	0	0	5138646	23209.389	86248.715	397312.71	200582.61	0	369162.15	19736.552	299616.81
POS	BisMePA(18:1/22:3)	BisMePA(40:4)+NH4	BisMePA	(18:1_22:3)	(18:1)	(22:3)		798.600733	C45 H85 O8 N1 P1	10779030	469845.95	4779417.2	1701417.3	942678.76	17017783	754229.75	12823717	7167873.8	1483042.6	25106628	38765264	700880.48
POS	BisMePA(18:2/18:2)	BisMePA(36:4)+NH4	BisMePA	(18:2_18:2)	(18:2)	(18:2)		742.538133	C41 H77 O8 N1 P1	513005463	984814.49	4686753	74201945	4296019.4	708562400	2148807.4	491206.7	619214.8	425460.07	10915972	41808526	73929.546
POS	Cer(d18:2/16:1)	Cer(d34:3)+H	Cer	(d18:2_16:1)	(d18:2)	(16:1)		534.488070	C34 H64 O3 N1	1038757.4	9133979	0	0	8093904.2	635507.07	151864.02	6859159.7	7356663.3	0	4631934.5	778215.19	1346168.5
POS	Cer(d34:1)	Cer(d34:1)+H	Cer	(d34:1)	(d34:1)			538.519370	C34 H68 O3 N1	19.614	20371902	0	0	18495932	121056.93	23044397	19510892	13892254	0	19916339	562232.86	660388.85
POS	DG(16:0/18:1)	DG(34:1)+NH4	DG	(16:0_18:1)	(16:0)	(18:1)		612.556150	C37 H74 O5 N1	2865617.7	437135720	889864.32	484952.67	292787111	2468034.2	170766634	296413190	203246597	251310.3	290697965	4251806.6	109399535
POS	DG(16:0/18:2)	DG(34:2)+Na	DG	(16:0_18:2)	(16:0)	(18:2)		615.495896	C37 H68 O5 Na1	8145126	2.098E+09	2663009	1187778.5	3.277E+09	33733806	2.254E+09	217039472	149894338	1199641.9	159522991	7909393.2	184659559
POS	DG(16:0/18:3)	DG(34:3)+H	DG	(16:0_18:3)	(16:0)	(18:3)		591.498301	C37 H67 O5	43510356	33257487	22049688	30080837	55240921	89185974	19076469	0	110911.12	0	207710.83	749644.67	97834.308
POS	DG(18:0/18:1)	DG(36:1)+NH4	DG	(18:0_18:1)	(18:0)	(18:1)		640.587450	C39 H78 O5 N1	36570015	18573718	258258.98	1195215.8	10820977	57356044	1263276.8	114004100	14212084	375393.69	62757639	89688487	307999.81
POS	DG(18:1/18:1)	DG(36:2)+NH4	DG	(18:1_18:1)	(18:1)	(18:1)		638.571800	C39 H76 O5 N1	6188986.8	2.682E+09	3230852.1	5186973.1	1.346E+09	14431482	2.099E+09	2.739E+09	2.584E+09	12194413	1.817E+09	119544140	2.423E+09
POS	DG(18:1/18:2)	DG(36:3)+NH4	DG	(18:1_18:2)	(18:1)	(18:2)		636.556150	C39 H74 O5 N1	25317698	3.769E+09	2491987.8	3593423	4.222E+09	19657511	1.136E+09	216514822	396283315	2869274.1	183423586	1807916.3	226378460
POS	DG(18:2/18:2)	DG(36:4)+NH4	DG	(18:2_18:2)	(18:2)	(18:2)		634.540500	C39 H72 O5 N1	2.197E+09	30536971	9624770.6	113003713	52830697	3.369E+09	12639811	49550515	7920443.9	6852719.6	49177360	44735584	3213566.6
POS	DG(18:3e/18:1)	DG(36:4e)+Na	DG	(18:3e_18:1)	(18:3e)	(18:1)		625.516631	C39 H70 O4 Na1	792444.95	864255044	91413.629	584879.94	710811473	4454823.2	363643950	2.246E+09	2.288E+09	601376.66	632468078	462379.93	2.023E+09
POS	DG(20:1/18:1)	DG(38:2)+Na	DG	(20:1_18:1)	(20:1)	(18:1)		671.558496	C41 H76 O5 Na1	20337459	19411675	548731.05	14011260	64251822	19242516	38310606	38182901	13633579	357800.97	44423754	99231322	6915120
POS	DG(20:1/18:2)	DG(38:3)+Na	DG	(20:1_18:2)	(20:1)	(18:2)		669.542846	C41 H74 O5 Na1	1071374.8	45745460	4020249.6	165489.89	101609267	324681.8	55591897	5917500.6	4148138	321683.96	5889104.7	648236.82	16587339
POS	DG(22:0/18:2)	DG(40:2)+Na	DG	(22:0_18:2)	(22:0)	(18:2)		699.589796	C43 H80 O5 Na1	42810241	1386238.8	3488007.4	12164530	87824.193	44645130	837003.2	4058353.4	621408.23	2683222.1	5808120.6	13765213	232849.12
POS	DG(32:1e)	DG(32:1e)+Na	DG	(32:1e)	(32:1e)			575.500981	C35 H68 O4 Na1	207964456	174501617	1752999.4	2356657.4	228767713	126439125	28704224	19731626	38895304	2397730.2	43184002	6933711.8	1387548.9
POS	DG(34:2e)	DG(34:2e)+NH4	DG	(34:2e)	(34:2e)			596.561235	C37 H74 O4 N1	108117.96	196340314	976051.65	244989.73	208203499	259397.03	195161734	307423030	291507190	915399.03	60394895	506877.62	81448552
POS	DG(34:3e)	DG(34:3e)+NH4	DG	(34:3e)	(34:3e)			594.545585	C37 H72 O4 N1	909307.06	60545124	0	0	65510094	570019.91	62707681	63039201	69626532	0	15287948	1155163.7	18360346
POS	DG(34:4e)	DG(34:4e)+H	DG	(34:4e)	(34:4e)			575.503386	C37 H67 O4	411860.02	64380515	408659.54	708864.43	155986740	260191.94	174645914	29754230	40990966	208050.49	13144850	9277900.7	46769413
POS	DG(36:0e)	DG(36:0e)+Na	DG	(36:0e)	(36:0e)			633.579231	C39 H78 O4 Na1	3951290.6	5305939.3	513028.99	4143259	32561125	16285130	27603299	42320747	52179892	72537.142	21458645	20834403	38751162
POS	DG(36:1e)	DG(36:1e)+Na	DG	(36:1e)	(36:1e)			631.563581	C39 H76 O4 Na1	4845622	5742616.4	52561.182	1342649	17518785	9473257	9006720.8	12643731	4921805.3	165530.87	12032887	18382549	1612203.2
POS	DG(36:3e)	DG(36:3e)+Na	DG	(36:3e)	(36:3e)			627.532281	C39 H72 O4 Na1	2869984.7	3.884E+09	1433208.5	1129005.8	3.547E+09	61464493	3.479E+09	2.292E+09	7.245E+09	1187559.1	1.708E+09	2033868.4	7.021E+09
POS	DG(36:4e)	DG(36:4e)+H	DG	(36:4e)	(36:4e)			603.534686	C39 H71 O4	116977679	1.551E+09	9600564.8	9859300.5	685156434	280855482	591595697	3.481E+09	1.823E+09	3816720.6	2.081E+09	523577054	363894914
POS	DG(36:5e)	DG(36:5e)+H	DG	(36:5e)	(36:5e)			601.519036	C39 H69 O4	583239844	418044563	11541292	6611357.7	335363299	934621161	50285665	169127129	92152537	371716.27	160318312	81690156	1312761.7
POS	DG(38:3e)	DG(38:3e)+H	DG	(38:3e)	(38:3e)			633.581636	C41 H77 O4	546912.96	5658304.7	150416.68	107498.72	33106685	3959712.2	30409984	34500624	48054171	326522.78	16327736	5356044.4	56177490
POS	Hex1Cer(d18:2/16:0)	Hex1Cer(d34:2+O)+H	Hex1Cer	(d18:2_16:0+O)	(d18:2)	(16:0+O)		714.551460	C40 H76 O9 N1	667224.34	28665330	40249.45	0	25336544	0	21716403	18546416	21248691	0	8216923.4	151699.39	24048832
POS	Hex1Cer(t18:1/22:0	Hex1Cer(t40:1+O)+H	Hex1Cer	(t18:1_22:0+O)	(t18:1)	(22:0+O)		816.655925	C46 H90 O10 N1	27490392	1881261.1	228862.99	1610007.8	421474.17	21164130	0	10452504	2492938.8	267091.35	12065535	17900220	91491.048
POS	Hex1Cer(t18:1/24:0)	Hex1Cer(t42:1+O)+H	Hex1Cer	(t18:1_24:0+O)	(t18:1)	(24:0+O)		844.687225	C48 H94 O10 N1	561325.85	42571139	129189.55	69590.604	15793889	253600.68	13662847	23771136	19356189	127421.75	15149895	334690.59	33355344
POS	LPC(18:0)	LPC(18:0)+H	LPC	(18:0)	(18:0)			524.371068	C26 H55 O7 N1 P1	5524396.7	134936.56	14224.846	344777.6	217488.24	8226595.8	44747.993	1491954	515630.69	18087.502	1025613.1	1122512.9	95881.437
POS	LPC(18:2)	LPC(18:2)+H	LPC	(18:2)	(18:2)			520.339768	C26 H51 O7 N1 P1	5082379	15637004	3685856.1	1087026.8	29796265	5773062	21843455	1838194.4	3743162.3	17591.295	55363.159	281333.61	8435688.7
POS	MePC(32:5)	MePC(32:5)+Na	MePC	(32:5)	(32:5)			760.488778	C41 H72 O8 N1 P1 Na1	0	10406183	0	0	25866933	0	13893409	605176.55	411735.91	0	262395.04	3.481	1026339.7
POS	MePC(33:1)	MePC(33:1)+Na	MePC	(33:1)	(33:1)			782.567028	C42 H82 O8 N1 P1 Na1	773057632	0	336175267	614854683	0	1.402E+09	262970.99	2625898.9	898981.11	215793.96	2907682.9	13871427	769734.61
POS	MePC(34:6)	MePC(34:6)+Na	MePC	(34:6)	(34:6)			786.504428	C43 H74 O8 N1 P1 Na1	0	16879066	0	0	16145883	0	15519318	1779179.6	3158593.8	0	395111.42	0	5263447.8
POS	MePC(34:7)	MePC(34:7)+Na	MePC	(34:7)	(34:7)			784.488778	C43 H72 O8 N1 P1 Na1	0	4858457.1	0	0	10553676	0	5021590.6	0	0	0	0	0	246797.08
POS	MGDG(18:1/18:3)	MGDG(36:4)+Na	MGDG	(18:1_18:3)	(18:1)	(18:3)		801.548721	C45 H78 O10 Na1	0	783168.06	0	0	2959241.4	0	1139373.6	185485.27	7796099.7	0	2036819.1	0	16349989
POS	PC(16:0/16:0)	PC(32:0)+Na	PC	(16:0_16:0)	(16:0)	(16:0)		756.551378	C40 H80 O8 N1 P1 Na1	226070.65	18735642	155898.27	110435.66	19460437	771719.32	9168327.6	10116467	2418969.6	0	207906.07	0	1940451.6
POS	PC(16:0/18:1)	PC(34:1)+H	PC	(16:0_18:1)	(16:0)	(18:1)		760.585128	C42 H82 O8 N1 P1 Na1	7792772.5	872208410	583345.64	1004765	612676005	15144524	597939626	579476990	727601152	506517.46	567583348	1992656.3	589896827
POS	PC(16:0/18:2)	PC(34:2)+Na	PC	(16:0_18:2)	(16:0)	(18:2)		780.551378	C42 H80 O8 N1 P1 Na1	1007381.2	393822589	101820.41	547269.23	610488761	1846013.6	357909071	30194637	26538949	152775.06	6882356.8	1335871.7	48119252
POS	PC(18:0/18:1)	PC(36:1)+Na	PC	(18:0_18:1)	(18:0)	(18:1)		810.598328	C44 H86 O8 N1 P1 Na1	915601.43	111874316	526632.02	564388.57	114794133	1172629.9	109997285	133460067	76227695	396988.8	38769841	428618.14	149195702
POS	PC(18:1/18:1)	PC(36:2)+Na	PC	(18:1_18:1)	(18:1)	(18:1)		808.582678	C44 H84 O8 N1 P1 Na1	1931010.1	1.349E+09	153053.5	2156449.3	562060256	10425507	871981008	2.462E+09	2.297E+09	3079086.9	1.036E+09	13890798	2.558E+09
POS	PC(18:1/18:2)	PC(36:3)+Na	PC	(18:1_18:2)	(18:1)	(18:2)		806.567028	C44 H82 O8 N1 P1 Na1	4196395.7	811885989	1383918.6	2308536.5	946054052	3388345.2	647772755	47409054	108063375	534546.84	21597509	151482.79	202443153
POS	PC(32:0)	PC(32:0)+H	PC	(16:0_16:0)	(16:0)	(16:0)		734.569433	C40 H81 O8 N1 P1	179536.58	91381892	549413.06	214999.12	96067780	193233.83	50536999	53203869	12975643	1437338.2	5067730	46480.056	9654271.5
POS	PC(32:1)	PC(32:1)+H	PC	(32:1)	(32:1)			732.553783	C40 H79 O8 N1 P1	244701.68	15691072	210533.29	103697.2	12187837	225233.5	7037389.3	8116688.1	10618574	113026.26	3245515.9	89833.536	10824263
POS	PC(32:2)	PC(32:2)+H	PC	(32:2)	(32:2)			730.538133	C40 H77 O8 N1 P1	12995451	1859647	256561.54	328228.22	289649.86	12391904	1132548.7	8651684	2601852.7	0	2609044.1	165767.91	789215.2
POS	PC(35:1)	PC(35:1)+H	PC	(35:1)	(35:1)			774.600733	C43 H85 O8 N1 P1	547060.14	24600803	169138.39	140468.18	22421055	478888.07	19622776	19036546	15383626	85371.454	6646397.9	297748.57	19721113
POS	PC(36:3)	PC(36:3)+H	PC	(18:1_18:2)	(18:1)	(18:2)		784.585083	C44 H83 O8 N1 P1	8497913.4	4.532E+09	1928239.2	6662397.1	4.891E+09	31969663	2.886E+09	520256330	762565761	152961.22	123629989	128287.08	1.097E+09
POS	PC(36:4)	PC(36:4)+H	PC	(18:2_18:2)	(18:2)	(18:2)		782.569433	C44 H81 O8 N1 P1	2.301E+09	25803179	3578227.3	10692754	32756144	2.862E+09	12141819	43385280	33810831	122197.22	28154743	3755418	3146555.6
POS	PC(38:2)	PC(38:2)+H	PC	(38:2)	(38:2)			814.632033	C46 H89 O8 N1 P1	11249918	2900945.3	425595.98	1971041.2	5167346	13375486	603274.01	13057599	6569438.3	648672.05	15929454	29364885	2436368.2
POS	PE(16:0/18:1)	PE(34:1)+H	PE	(16:0_18:1)	(16:0)	(18:1)		718.538133	C39 H77 O8 N1 P1	1168425.5	551239339	819060.05	867402.06	451048445	4932960.3	456483672	316818770	462189665	2274850.8	178241832	1717485.2	510560072
POS	PE(16:0/18:2)	PE(34:2)+H	PE	(16:0_18:2)	(16:0)	(18:2)		716.522483	C39 H75 O8 N1 P1	2094979.8	322350742	1420346.8	684591.22	732072069	8074475.4	391845168	40333309	39688602	92965.537	14501227	714396.89	65529324
POS	PE(18:0/18:1)	PE(36:1)+H	PE	(18:0_18:1)	(18:0)	(18:1)		746.569433	C41 H81 O8 N1 P1	426511.75	33271098	153806.21	0	26481105	1065360	26231837	48809029	23714671	579486.67	15714781	472876.7	27157401
POS	PE(18:1/18:1)	PE(36:2)+H	PE	(18:1_18:1)	(18:1)	(18:1)		744.553783	C41 H79 O8 N1 P1	728431.95	609174764	1496140	2151521.4	194520016	9413498	311207933	1.239E+09	1.252E+09	1358678.3	763597480	14068583	465580805
POS	PE(18:1/18:2)	PE(36:3)+H	PE	(18:1_18:2)	(18:1)	(18:2)		742.538133	C41 H77 O8 N1 P1	1479320.8	513912377	135529.75	109323.03	500626924	1176668.5	409183128	48957839	84030896	222154.61	23824808	0	117990004
POS	PE(34:1)	PE(34:1)+Na	PE	(16:0_18:1)	(16:0)	(18:1)		740.520078	C39 H76 O8 N1 P1 Na1	489385.93	84563523	606353.02	167824.64	80358302	836394.25	82019599	53578348	79939391	94799.378	31272026	251414.14	87978411
POS	PE(36:1)	PE(36:1)+Na	PE	(18:0_18:1)	(18:0)	(18:1)		768.551378	C41 H80 O8 N1 P1 Na1	10161102	460229.86	366600.13	124928.86	173391.06	10545463	130007.47	3139342.2	183863.73	236055.39	7203459.9	13997629	45118.925
POS	PE(36:3)	PE(36:3)+Na	PE	(18:1_18:2)	(18:1)	(18:2)		764.520078	C41 H76 O8 N1 P1 Na1	505116.31	0	42901661	7458780.2	0	144693.6	0	0	0	16815299	0	0	0
POS	PE(36:4)	PE(36:4)+Na	PE	(18:2_18:2)	(18:2)	(18:2)		762.504428	C41 H74 O8 N1 P1 Na1	46167520	124966.04	51178.875	568053.54	97982.935	54213195	0	156049.86	0	0	0	106862.12	0
POS	PG(28:0/18:1)	PG(46:1)+H	PG	(28:0_18:1)	(28:0)	(18:1)		917.720514	C52 H102 O10 N0 P1	2.149E+09	409327067	271583014	322372845	439408617	2.362E+09	199507358	12456753	21651502	7462417.3	22736700	27513986	5709261.4
POS	PI(18:1/18:1)	PI(36:2)+H	PI	(18:1_18:1)	(18:1)	(18:1)		863.564409	C45 H84 O13 N0 P1	106593.07	2541496.6	0	26378.9	874011.49	31380.894	4789952.8	15653383	20940422	47058.304	14365727	39694.896	744922.22
POS	PI(36:1)	PI(36:1)+H	PI	(36:1)	(36:1)			865.580059	C45 H86 O13 N0 P1	7789569.6	0	0	0	0	12018090	0	0	0	0	0	3645224.8	0
POS	PI(36:3)	PI(36:3)+NH4	PI	(18:1_18:2)	(18:1)	(18:2)		878.575308	C45 H85 O13 N1 P1	8892997.7	0	468987.05	93141.767	0	12951931	0	0	0	33496.064	0	135474.53	0
POS	TG(10:0/10:1/18:1)	TG(38:2)+Na	TG	(10:0_10:1_18:1)	(10:0)	(10:1)	(18:1)	685.537761	C41 H74 O6 Na1	0	43346352	0	103341.63	20086766	295656.7	17558667	104772083	153469794	1203288.6	53113037	1265408.4	123340220
POS	TG(12:1e/6:0/18:3)	TG(36:4e)+H	TG	(12:1e_6:0_18:3)	(12:1e)	(6:0)	(18:3)	617.513951	C39 H69 O5	15467114	2.654E+09	5431127.9	7784526.8	893133129	5064882.3	1.829E+09	907643705	1.321E+09	2185056.4	586441792	77325334	899674017
POS	TG(14:0/18:2/18:2)	TG(50:4)+Na	TG	(14:0_18:2_18:2)	(14:0)	(18:2)	(18:2)	849.694261	C53 H94 O6 Na1	159846368	21465732	28224878	125556574	16943051	143350579	5461665.1	11758132	16080863	140709.76	5598952.2	3807204.9	7279558.7
POS	TG(15:0/18:1/20:5)	TG(53:6)+H	TG	(15:0_18:1_20:5)	(15:0)	(18:1)	(20:5)	865.727966	C56 H97 O6	1887526.8	55072545	252904.49	0	141209316	8447847	105943387	1836316	12529446	228648.91	6697858.7	2328438.1	53877136
POS	TG(15:0/18:2/18:2)	TG(51:4)+NH4	TG	(15:0_18:2_18:2)	(15:0)	(18:2)	(18:2)	858.754515	C54 H100 O6 N1	35529885	5313728.1	15076414	45810808	13606602	23849992	9549284.4	843088.65	3556033.5	0	941175.47	1124050.8	424489.05
POS	TG(16:0/14:0/18:1)	TG(48:1)+NH4	TG	(16:0_14:0_18:1)	(16:0)	(14:0)	(18:1)	822.754515	C51 H100 O6 N1	17520706	3252622.5	165657.74	20828875	3871962.6	21974480	523102.14	15079281	8850617.8	254854.09	14171973	10099557	554343.21
POS	TG(16:0/14:0/18:2)	TG(48:2)+NH4	TG	(16:0_14:0_18:2)	(16:0)	(14:0)	(18:2)	820.738865	C51 H98 O6 N1	52227295	3275188.9	40137235	56466605	1016282.9	52028230	52312.423	6972843	2558197.5	15170177	9025575.6	1934077.3	1096610.1
POS	TG(16:0/14:0/22:6)	TG(52:6)+Na	TG	(16:0_14:0_22:6)	(16:0)	(14:0)	(22:6)	873.694335	C55 H95 O6	119936.05	30259981	0	788030.89	37083363	730082.16	18729517	0	294626.04	0	178541.3	0	238408.19
POS	TG(16:0/16:0/18:1)	TG(50:1)+Na	TG	(16:0_16:0_18:1)	(16:0)	(16:0)	(18:1)	855.741211	C53 H100 O6 Na1	62452316	152363398	5649645	2816163.7	430104160	162318824	382075722	116268499	110807289	4801316.9	86185925	66812633	109424982
POS	TG(16:0/16:0/18:2)	TG(50:2)+NH4	TG	(16:0_16:0_18:2)	(16:0)	(16:0)	(18:2)	848.770165	C53 H102 O6 N1	996531062	4870131.2	714800143	945188005	78160095	942943720	253073304	64310773	64231884	275573328	85438834	65947118	21433065
POS	TG(16:0/16:0/18:3)	TG(50:3)+Na	TG	(16:0_16:0_18:3)	(16:0)	(16:0)	(18:3)	851.709911	C53 H96 O6 Na1	381858.8	30048016	0	845866.88	37538004	730082.16	28007674	0	292739.79	0	0	0	258767.73
POS	TG(16:0/17:0/18:1)	TG(51:1)+Na	TG	(16:0_17:0_18:1)	(16:0)	(17:0)	(18:1)	869.756861	C54 H102 O6 Na1	2794974.9	10709942	0	0	45054252	18838617	26081896	15501121	21459540	0	12972545	11214767	25950344
POS	TG(16:0/17:1/18:1)	TG(51:2)+Na	TG	(16:0_17:1_18:1)	(16:0)	(17:1)	(18:1)	867.741211	C54 H100 O6 Na1	11311768	49441899	0	1521721.7	153911896	21866868	117945539	65933097	88136141	233299.6	43199196	45211386	47269942
POS	TG(16:0/17:1/18:2)	TG(51:3)+Na	TG	(16:0_17:1_18:2)	(16:0)	(17:1)	(18:2)	865.725561	C54 H98 O6 Na1	49782679	21660967	21193768	50499193	35573809	55671641	20113480	22034463	35978128	140455.69	21543843	11947783	53841494
POS	TG(16:0/18:1/18:1)	TG(52:2)+Na	TG	(16:0_18:1_18:1)	(16:0)	(18:1)	(18:1)	881.756861	C55 H102 O6 Na1	473663674	803468070	45705166	42173569	679470053	2.081E+09	1.078E+09	1.828E+09	701299596	13255360	1.597E+09	1.463E+09	451911617
POS	TG(16:0/18:1/18:3)	TG(52:4)+H	TG	(16:0_18:1_18:3)	(16:0)	(18:1)	(18:3)	855.743616	C55 H99 O6	888713232	426150507	12251876	480567177	363340774	2.775E+09	915977867	3608385.1	6843876.7	172356.75	5657704.4	36991798	13470176
POS	TG(16:0/18:1/22:0)	TG(56:1)+Na	TG	(16:0_18:1_22:0)	(16:0)	(18:1)	(22:0)	939.835111	C59 H112 O6 Na1	29494089	23209826	339574.09	431261.63	301059519	98544502	222709772	111643047	133814630	2892189.1	61503674	52083732	132063093
POS	TG(16:0/18:1/24:0)	TG(58:1)+NH4	TG	(16:0_18:1_24:0)	(16:0)	(18:1)	(24:0)	962.911015	C61 H120 O6 N1	7108463.2	5127328.4	408153.38	14451690	165606708	55148692	113342102	88715701	141227620	42276.935	31263634	34034189	129753537
POS	TG(16:0/18:2/18:2)	TG(52:4)+NH4	TG	(16:0_18:2_18:2)	(16:0)	(18:2)	(18:2)	872.770165	C55 H102 O6 N1	3221370.7	2025590.7	3442070.8	1742975.6	3606310.9	2893518.7	18096865	0	0	0	0	109976.47	253811.88
POS	TG(16:0/18:2/20:4)	TG(54:6)+H	TG	(16:0_18:2_20:4)	(16:0)	(18:2)	(20:4)	879.743616	C57 H99 O6	2.795E+09	28546115	2.472E+09	2.628E+09	472017615	685557835	1.149E+09	65381094	92356906	1.298E+09	141820816	341407442	65258394
POS	TG(16:0/20:4/24:0)	TG(60:4)+H	TG	(16:0_20:4_24:0)	(16:0)	(20:4)	(24:0)	967.868816	C63 H115 O6	4050452.6	5958135.4	625276.13	897219.74	129001919	27936856	94111957	53128386	78778544	5987481.3	26441280	14337519	108258878
POS	TG(16:0/8:0/10:0)	TG(34:0)+NH4	TG	(16:0_8:0_10:0)	(16:0)	(8:0)	(10:0)	628.551065	C37 H74 O6 N1	1155741.3	31709727	97179.054	274607.27	36732359	868773.23	114321499	756821.83	1372568	0	185074.34	0	3887643.2
POS	TG(16:1/18:1/18:1)	TG(52:3)+NH4	TG	(16:1_18:1_18:1)	(16:1)	(18:1)	(18:1)	874.785815	C55 H104 O6 N1	3.777E+09	242624977	3.358E+09	4.236E+09	254323013	4.386E+09	1.572E+09	222614359	319200955	1.598E+09	542149336	1.674E+09	75644179
POS	TG(16:1/18:2/18:2)	TG(52:5)+NH4	TG	(16:1_18:2_18:2)	(16:1)	(18:2)	(18:2)	870.754515	C55 H100 O6 N1	240235799	99451996	1005455.3	162153484	58703849	392151501	70462665	11441488	23771258	0	14656032	2507932.5	136259689
POS	TG(17:0/18:1/18:1)	TG(53:2)+NH4	TG	(17:0_18:1_18:1)	(17:0)	(18:1)	(18:1)	890.817115	C56 H108 O6 N1	18664962	47328696	441933.15	534757.49	250038098	65848976	175818162	195042456	223338956	3578680.9	107556971	108054415	201696920
POS	TG(17:0/18:1/20:4)	TG(55:5)+H	TG	(17:0_18:1_20:4)	(17:0)	(18:1)	(20:4)	895.774916	C58 H103 O6	38172292	57958265	3051742.1	13505978	235694428	101906940	128211901	162839853	220380641	281311.15	128177465	118712290	143614774
POS	TG(17:0/18:1/20:5)	TG(55:6)+NH4	TG	(17:0_18:1_20:5)	(17:0)	(18:1)	(20:5)	910.785875	C58 H101 O6	127172911	23866771	237237421	218151495	141022685	233283677	50034771	22411367	24626752	116252791	43509775	219280857	1369811.1
POS	TG(17:0/18:2/18:3)	TG(53:5)+H	TG	(17:0_18:2_18:3)	(17:0)	(18:2)	(18:3)	867.743616	C56 H99 O6	46463670	162396.61	84178311	74924314	36323053	64625705	8129282.3	4528772.6	5620825.9	34998706	9787572.5	30544468	534851.7
POS	TG(18:0/16:0/18:1)	TG(52:1)+NH4	TG	(18:0_16:0_18:1)	(18:0)	(16:0)	(18:1)	878.817115	C55 H108 O6 N1	236347636	191417111	145634.11	192125.37	935873528	468796786	698257099	368300990	325448718	4446291.9	269973843	237298735	272066173

POS	TG(18:0/17:0/18:1)	TG(53:1)+NH4	TG	(18:0_17:0_18:1)	(18:0)	(17:0)	(18:1)	892.832765	C56 H110 O6 N1	1006486.7	1837796.5	7242.2376	761641.6	21438556	4419967.2	14525109	12682795	7224374.9	0	4069709.4	5076698.3	13331888
POS	TG(18:0/18:1/18:1)	TG(54:2)+NH4	TG	(18:0_18:1_18:1)	(18:0)	(18:1)	(18:1)	904.832765	C57 H110 O6 N1	1.01E+09	905322274	66988166	284922585	2.935E+09	1.979E+09	2.26E+09	3.218E+09	2.933E+09	3509241.8	2.216E+09	2.222E+09	2.568E+09
POS	TG(18:0/18:1/18:3)	TG(54:4)+H	TG	(18:0_18:1_18:3)	(18:0)	(18:1)	(18:3)	883.774916	C57 H103 O6	699700.53	569138773	31654753	38259386	1.017E+09	13913418	744822250	64657483	67469507	64089243	63058686	23382203	496232
POS	TG(18:0/18:1/24:0)	TG(60:1)+NH4	TG	(18:0_18:1_24:0)	(18:0)	(18:1)	(24:0)	990.942315	C63 H124 O6 N1	479472.15	314463.35	0	1430524.1	23124477	9151813.3	13623259	21245010	34784506	952949.09	5002610.2	3508607.6	35136176
POS	TG(18:1/10:1/10:1)	TG(38:3)+Na	TG	(18:1_10:1_10:1)	(18:1)	(10:1)	(10:1)	683.522112	C41 H72 O6 Na1	234283.8	39615152	0	0	45990627	238861.29	41809365	2453584.1	8469310.6	0	1156714	0	19268983
POS	TG(18:1/14:0/18:2)	TG(50:3)+NH4	TG	(18:1_14:0_18:2)	(18:1)	(14:0)	(18:2)	846.754515	C53 H100 O6 N1	241389847	3705065.2	197707917	320560971	2879351.2	309712940	3050222.1	97212440	20668226	1171126.6	97750494	61347200	40686508
POS	TG(18:1/14:1/20:4)	TG(52:6)+H	TG	(18:1_14:1_20:4)	(18:1)	(14:1)	(20:4)	851.712316	C55 H95 O6	14588392	2726700.8	267417103	246032877	3207835.6	11011733	1495011.3	48486639	19635510	95473371	31267691	10526184	2159528.1
POS	TG(18:1/17:1/18:1)	TG(53:3)+Na	TG	(18:1_17:1_18:1)	(18:1)	(17:1)	(18:1)	893.756861	C56 H102 O6 Na1	113977504	10606206	236705063	219860765	25808765	212510288	9250831.9	12232226	6519154.9	116708411	12128438	60869813	532028.76
POS	TG(18:1/17:1/18:2)	TG(53:4)+Na	TG	(18:1_17:1_18:2)	(18:1)	(17:1)	(18:2)	891.741211	C56 H100 O6 Na1	43586400	136417150	3865185	19883040	624556336	108745723	432976681	21902486	41298004	0	19874461	16508848	8554302.8
POS	TG(18:1/17:1/20:4)	TG(55:6)+H	TG	(18:1_17:1_20:4)	(18:1)	(17:1)	(20:4)	893.759266	C58 H101 O6	51975980	198364027	10347520	19452952	588359950	233240410	344931538	269783495	251286982	3111464.7	159382369	218461627	80349077
POS	TG(18:1/18:1/18:1)	TG(54:3)+NH4	TG	(18:1_18:1_18:1)	(18:1)	(18:1)	(18:1)	902.817115	C57 H108 O6 N1	1.205E+09	4.723E+09	80948989	158794519	1.63E+09	2.625E+09	3.998E+09	1.053E+10	1.541E+10	27128670	6.621E+09	4.646E+09	6.434E+09
POS	TG(18:1/18:1/18:2)	TG(54:4)+NH4	TG	(18:1_18:1_18:2)	(18:1)	(18:1)	(18:2)	900.801465	C57 H106 O6 N1	8.282E+09	586317878	7.624E+09	8.885E+09	1.4E+09	1.037E+10	1.385E+09	483052490	640420086	2.464E+09	1.228E+09	3.895E+09	154665734
POS	TG(18:1/18:1/18:3)	TG(54:5)+H	TG	(18:1_18:1_18:3)	(18:1)	(18:1)	(18:3)	881.759266	C57 H101 O6	55692773	954046034	735551846	4.577E+09	922712221	570922830	815727460	16566328	36514995	6806919.6	56671416	169960517	20187250
POS	TG(18:1/18:1/20:3)	TG(56:5)+H	TG	(18:1_18:1_20:3)	(18:1)	(18:1)	(20:3)	909.790566	C59 H105 O6	940869469	315963554	1.097E+09	1.248E+09	616948327	1.164E+09	228912302	577083012	915957163	1.434E+09	758809714	1.024E+09	418526683
POS	TG(18:1/18:1/20:4)	TG(56:6)+H	TG	(18:1_18:1_20:4)	(18:1)	(18:1)	(20:4)	907.774916	C59 H103 O6	3.576E+09	4.149E+09	119651690	14707460	3.637E+09	3.847E+09	1.065E+09	7.776E+09	7.947E+09	331779416	6.72E+09	5.924E+09	8.128E+09
POS	TG(18:1/18:1/20:5)	TG(56:7)+H	TG	(18:1_18:1_20:5)	(18:1)	(18:1)	(20:5)	905.759266	C59 H101 O6	22001318	32504518	31017017	16576316	56719579	52138781	122420968	4058554.5	8061040.5	9621620.8	6653259.9	5081347.9	8927693.3
POS	TG(18:1/18:1/21:0)	TG(57:2)+NH4	TG	(18:1_18:1_21:0)	(18:1)	(18:1)	(21:0)	946.879715	C60 H116 O6 N1	303587.59	2016936.7	384111.34	408242.5	34702129	4663932.5	27297225	14735810	27724623	651914.68	7167737.2	5978238.4	29606545
POS	TG(18:1/18:1/22:0)	TG(58:2)+NH4	TG	(18:1_18:1_22:0)	(18:1)	(18:1)	(22:0)	960.895365	C61 H118 O6 N1	59104459	65990628	376930.82	27789.944	962016740	290693559	723555767	975771772	1.615E+09	2513987.1	461150136	377023931	1.156E+09
POS	TG(18:1/18:1/23:0)	TG(59:2)+Na	TG	(18:1_18:1_23:0)	(18:1)	(18:1)	(23:0)	979.866411	C62 H116 O6 Na1	238691.49	222675.56	3.861	383470.24	23521117	3605712.2	17780293	17732195	29176661	0	6432083.4	4981496.4	33983971
POS	TG(18:1/18:1/24:0)	TG(60:2)+Na	TG	(18:1_18:1_24:0)	(18:1)	(18:1)	(24:0)	993.882061	C63 H118 O6 Na1	17556117	18370827	1508091.1	26983586	248973761	111945357	157518534	314744916	514985280	161501.9	148880336	105273345	383258299
POS	TG(18:1/18:2/18:2)	TG(54:5)+NH4	TG	(18:1_18:2_18:2)	(18:1)	(18:2)	(18:2)	898.785815	C57 H104 O6 N1	1.413E+09	7.62E+09	95836105	239872442	9.792E+09	3.039E+09	7.977E+09	1.025E+09	2.231E+09	1766454.7	760611554	239864282	2.019E+09
POS	TG(18:1/18:2/20:5)	TG(56:8)+H	TG	(18:1_18:2_20:5)	(18:1)	(18:2)	(20:5)	903.743616	C59 H99 O6	6.462E+09	3.121E+09	322681141	1.097E+09	3.708E+09	7.347E+09	1.042E+09	1.091E+09	1.081E+09	6010412.1	1.24E+09	1.233E+09	766934261
POS	TG(18:1/18:2/21:0)	TG(57:3)+Na	TG	(18:1_18:2_21:0)	(18:1)	(18:2)	(21:0)	949.819461	C60 H110 O6 Na1	206829.25	5434298.9	0	616310.48	60661531	8228325.5	43277731	743465.11	27094683	218115.14	2008098.9	2616279.5	8376084.7
POS	TG(18:1/18:2/22:0)	TG(58:3)+Na	TG	(18:1_18:2_22:0)	(18:1)	(18:2)	(22:0)	963.835111	C61 H112 O6 Na1	296524362	190893683	15219883	6292670.2	447417412	554063191	637967743	191796171	472324601	44411915	128719210	124277105	355376163
POS	TG(18:1/18:2/23:0)	TG(59:3)+Na	TG	(18:1_18:2_23:0)	(18:1)	(18:2)	(23:0)	977.850761	C62 H114 O6 Na1	2984078.3	1994043.8	0	404932.45	44931467	12842394	34047218	1222432	6143657.6	0	1239892.6	978580.82	9441475.2
POS	TG(18:1/18:2/24:0)	TG(60:3)+Na	TG	(18:1_18:2_24:0)	(18:1)	(18:2)	(24:0)	991.866411	C63 H116 O6 Na1	52630720	37421520	460759.13	35491220	344042543	153382350	240061021	38546456	117624509	2738239.7	26335259	18261959	118107498
POS	TG(18:1/20:3/24:0)	TG(62:4)+H	TG	(18:1_20:3_24:0)	(18:1)	(20:3)	(24:0)	995.900116	C65 H119 O6	158714.23	388614.65	0	105421.69	25706954	6667957.9	13264461	16263470	27657673	1842861.5	4480074.4	3396865	32587402
POS	TG(18:2/14:1/18:2)	TG(50:5)+Na	TG	(18:2_14:1_18:2)	(18:2)	(14:1)	(18:2)	847.678611	C53 H92 O6 Na1	9558878.4	24027347	263666.25	370673.05	36414331	18948161	25093695	436892.03	181281.33	0	156291.29	0	208901.96
POS	TG(18:2/17:1/18:2)	TG(53:5)+Na	TG	(18:2_17:1_18:2)	(18:2)	(17:1)	(18:2)	889.725561	C56 H98 O6 Na1	51388339	218408.69	21956320	62455253	26166969	93715815	11571953	713337.38	1990553	0	203103.71	529658.51	746390.8
POS	TG(18:2/18:2/18:2)	TG(54:6)+NH4	TG	(18:2_18:2_18:2)	(18:2)	(18:2)	(18:2)	896.770165	C57 H102 O6 N1	6.392E+09	333592855	5.2E+09	7.925E+09	1.177E+09	6.136E+09	509770230	35009764	89225998	990170.82	58893756	80442527	12017221
POS	TG(18:2/18:2/21:0)	TG(57:4)+Na	TG	(18:2_18:2_21:0)	(18:2)	(18:2)	(21:0)	947.803811	C60 H108 O6 Na1	314822.92	4735289.5	0	1310429.1	41170406	1512461.6	30156916	0	2270813.1	0	0	0	708230.59
POS	TG(18:2/18:2/23:0)	TG(59:4)+Na	TG	(18:2_18:2_23:0)	(18:2)	(18:2)	(23:0)	975.835111	C62 H112 O6 Na1	1212167	2357524	0	0	41722355	5453184.9	37710831	0	938183.02	0	0	0	1820703.3
POS	TG(18:2/20:4/21:0)	TG(59:6)+H	TG	(18:2_20:4_21:0)	(18:2)	(20:4)	(21:0)	949.821866	C62 H109 O6	6521283.8	5441970.5	1875598.9	14117795	60735286	14514142	43154058	2076929.4	27422389	1410174.7	1066456.9	2519230.1	2972118.4
POS	TG(18:3/18:2/18:2)	TG(54:7)+NH4	TG	(18:3_18:2_18:2)	(18:3)	(18:2)	(18:2)	894.754515	C57 H100 O6 N1	130650806	410661.1	24021095	113176398	1902645.9	71742038	3444271.3	824431.74	1033240.7	85868.382	3190653.4	4877802.5	308813.68
POS	TG(19:0/18:1/18:1)	TG(55:2)+NH4	TG	(19:0_18:1_18:1)	(19:0)	(18:1)	(18:1)	918.848415	C58 H112 O6 N1	342823.73	3106071.6	1015103.5	112641.01	47703517	4813763	41592347	21182460	20681051	1810403.2	7982467.6	6978774.1	22933258
POS	TG(19:1/18:1/18:1)	TG(55:3)+Na	TG	(19:1_18:1_18:1)	(19:1)	(18:1)	(18:1)	921.788161	C58 H106 O6 Na1	6425658.7	26698381	332110.09	2033485.4	120661282	42917054	72650787	70770716	104900760	0	51723716	43303998	73347062
POS	TG(19:1/18:1/18:2)	TG(55:4)+Na	TG	(19:1_18:1_18:2)	(19:1)	(18:1)	(18:2)	919.772511	C58 H104 O6 Na1	29644832	38385826	558908.01	2760239.9	157657118	62256001	82697126	4723991.1	9683320.8	0	3772085.1	3913096.3	5957386.6
POS	TG(19:1/18:2/18:2)	TG(55:5)+Na	TG	(19:1_18:2_18:2)	(19:1)	(18:2)	(18:2)	917.756861	C58 H102 O6 Na1	22558657	3401421	12682527	35101226	22298651	41286607	5214802.5	0	1130991.6	0	188904.91	0	131733.01
POS	TG(20:0/16:0/18:1)	TG(54:1)+Na	TG	(20:0_16:0_18:1)	(20:0)	(16:0)	(18:1)	911.803811	C57 H108 O6 Na1	48540372	60494611	124146.11	639953.1	193490899	146405744	282299170	211562751	162253902	4895394.7	101289472	118846039	197964557
POS	TG(20:0/18:1/18:1)	TG(56:2)+NH4	TG	(20:0_18:1_18:1)	(20:0)	(18:1)	(18:1)	932.864065	C59 H114 O6 N1	285190687	17363825	255153920	635293661	195961576	804007160	145081240	216904414	280728436	547950313	192425924	345332845	41862948
POS	TG(20:0/18:1/20:4)	TG(58:5)+H	TG	(20:0_18:1_20:4)	(20:0)	(18:1)	(20:4)	937.821866	C61 H109 O6	124038918	215473850	1037910.3	114608813	590239600	623663226	445955174	911106465	1.02E+09	7148356.4	560541676	592645780	863281841
POS	TG(20:0/18:2/20:5)	TG(58:7)+H	TG	(20:0_18:2_20:5)	(20:0)	(18:2)	(20:5)	933.790566	C61 H105 O6	819789138	122081050	555027540	881406725	619652324	1.056E+09	181745644	17714773	70094370	144626176	35497175	19263473	10249958
POS	TG(20:1/18:1/18:1)	TG(56:3)+NH4	TG	(20:1_18:1_18:1)	(20:1)	(18:1)	(18:1)	930.848415	C59 H112 O6 N1	91987176	433275994	2148049.5	471688.75	1.13E+09	184885949	1.178E+09	1.003E+09	2.126E+09	8907118	853518306	538284813	1.256E+09
POS	TG(20:1/18:1/18:2)	TG(56:4)+NH4	TG	(20:1_18:1_18:2)	(20:1)	(18:1)	(18:2)	928.832765	C59 H110 O6 N1	286485906	341969265	53793396	236327030	2.332E+09	253898714	1.986E+09	189005211	256733101	10215622	172727768	119924093	145756714
POS	TG(20:1/18:1/20:4)	TG(58:6)+H	TG	(20:1_18:1_20:4)	(20:1)	(18:1)	(20:4)	935.806216	C61 H107 O6	483359502	194934933	99219035	687361854	352094340	741916439	10216557	152488691	315648992	780041492	172202956	124761278	109361143
POS	TG(20:1/18:2/18:2)	TG(56:5)+NH4	TG	(20:1_18:2_18:2)	(20:1)	(18:2)	(18:2)	926.817115	C59 H108 O6 N1	488590421	4318725.3	266952798	530128347	102267917	499172634	49213076	19840945	42342359	31426220	23214882	64111947	5062109.9
POS	TG(20:5/14:1/18:2)	TG(52:8)+H	TG	(20:5_14:1_18:2)	(20:5)	(14:1)	(18:2)	847.681016	C55 H91 O6	7489231.9	23092663	281226.58	395344.28	26749426	16787395	37065037	437026.28	197056.72	0	156602.02	0	1501986.8
POS	TG(20:5/17:1/18:2)	TG(55:8)+H	TG	(20:5_17:1_18:2)	(20:5)	(17:1)	(18:2)	889.727966	C58 H97 O6	0	28711208	241900.91	0	272174602	231916.62	212653906	453938.75	1760875.3	0	138362.67	0	6926083.4
POS	TG(22:0/18:2/18:2)	TG(58:4)+NH4	TG	(22:0_18:2_18:2)	(22:0)	(18:2)	(18:2)	956.864065	C61 H114 O6 N1	502239557	29890389	177425127	722707382	538236750	884362732	258715631	57925155	118749079	19121653	42047580	43408455	11694381
POS	TG(22:0/18:2/20:5)	TG(60:7)+H	TG	(22:0_18:2_20:5)	(22:0)	(18:2)	(20:5)	961.821866	C63 H109 O6	590149848	98552119	302132245	718645027	764122627	804680622	406369517	49890715	206451308	13594477	34162180	41908481	51026727
POS	TG(22:1/18:2/18:2)	TG(58:5)+NH4	TG	(22:1_18:2_18:2)	(22:1)	(18:2)	(18:2)	954.848415	C61 H112 O6 N1	30513117	435517.23	16317438	33720250	20527638	25133354	20338396	341973.97	2895561.7	1988561.9	674219.89	2048013.9	8593381.4
POS	TG(24:0/18:2/18:2)	TG(60:4)+NH4	TG	(24:0_18:2_18:2)	(24:0)	(18:2)	(18:2)	984.895365	C63 H118 O6 N1	102454498	38888804	4046314	59955465	594466816	232231019	522617683	8025171.2	36720736	326319.76	4555059.4	4969846.1	51446970
POS	TG(24:0/18:2/18:3)	TG(60:5)+H	TG	(24:0_18:2_18:3)	(24:0)	(18:2)	(18:3)	965.853166	C63 H113 O6	11376799	668097.59	224427.11	4364033.8	5428701.8	22333389	44082956	34828.724	229215.34	0	52102.307	0	4.217
POS	TG(26:0/18:1/18:1)	TG(62:2)+Na	TG	(26:0_18:1_18:1)	(26:0)	(18:1)	(18:1)	1021.913361	C65 H122 O6 Na1	964679.97	99097.08	426765.9	15465616	6921205.1	10494145	3363288.8	24509733	59596183	10361785	11138284	8264094.3	7782103.6
POS	TG(26:0/18:1/18:2)	TG(62:3)+NH4	TG	(26:0_18:1_18:2)	(26:0)	(18:1)	(18:2)	1014.942315	C65 H124 O6 N1	2557203.7	1404795.3	0	3704903	50695451	20629588	32690277	4360860.8	16592226	0	2029114.5	1021887.6	24365578
POS	TG(26:0/18:2/18:2)	TG(62:4)+NH4	TG	(26:0_18:2_18:2)	(26:0)	(18:2)	(18:2)	1012.926665	C65 H122 O6 N1	4849497.4	1854704.5	88963.884	3924670.5	63260825	25663820	45324799	0	1510258.8	0	0	0	6021298.9
POS	TG(37:1e)	TG(37:1e)+NH4	TG	(37:1e)	(37:1e)			654.603100	C40 H80 O5 N1	39109677	5887008.9	2032106.8	14529704	2973847.4	61221439	4734586.6	14804908	2340267.1	0	62215134	63106833	2508019.8
POS	TG(38:5e)	TG(38:5e)+H	TG	(38:5e)	(38:5e)			643.529601	C41 H71 O5	49892787	7.24E+09	5252239.2	15899461	3.962E+09	6018798.6	5.858E+09	1.035E+10	7.677E+09	13856109	2.949E+09	674157640	7.784E+09
POS	TG(39:2e)	TG(39:2e)+NH4	TG	(39:2e)	(39:2e)			680.618750	C42 H82 O5 N1	342357.63	751599837	113828.07	477924.29	762682980	15399085	279024895	932144636	982203917	41369.432	255965743	18521943	209031421
POS	TG(42:1)	TG(42:1)+Na	TG	(42:1)	(42:1)			743.616011	C45 H84 O6 Na1	255534.12	88733551	0	83447.4	81834719	6666620.6	88789147	109160626	93146748	267222.35	24511342	14670128	19734509
POS	TG(6:0/6:0/20:4)	TG(32:4)+Na	TG	(6:0_6:0_20:4)	(6:0)	(6:0)	(20:4)	597.412561	C35 H58 O6 Na1	198163.76	31413722	86011.457	58434.899	21257372	697643.92	11462515	24414924	6808083.5	127017.3	6834959.8	277428.6	9593903.6
POS	TG(9:0/12:0/18:1)	TG(39:1)+NH4	TG	(9:0_12:0_18:1)	(9:0)	(12:0)	(18:1)	696.613665	C42 H82 O6 N1	9596709.5	22126015	220634.53	611122.52	18009790	16079287	1756756.7	12773337	14981175	190077.97	71170717	2069618.5	3034824
POS	TG(9:0/9:0/18:1)	TG(36:1)+H	TG	(9:0_9:0_18:1)	(9:0)	(9:0)	(18:1)	637.540166	C39 H73 O6	384301.89	94354856	319118.61	584631.17	72971609	4803158.7	74070949	1788385	3131630	447077.52	1201651.8	816249.46	4927880.2
