# Supplementary figures and images for: A Comparison of Lipid Contents in Different Types of Peanut Cultivars Using UPLC-Q-TOF-MS-Based Lipidomic Study
Source: Foods. 2021 Dec 21;11(1):4. doi: 10.3390/foods11010004 (PMC8750182; doi:10.3390/foods11010004)

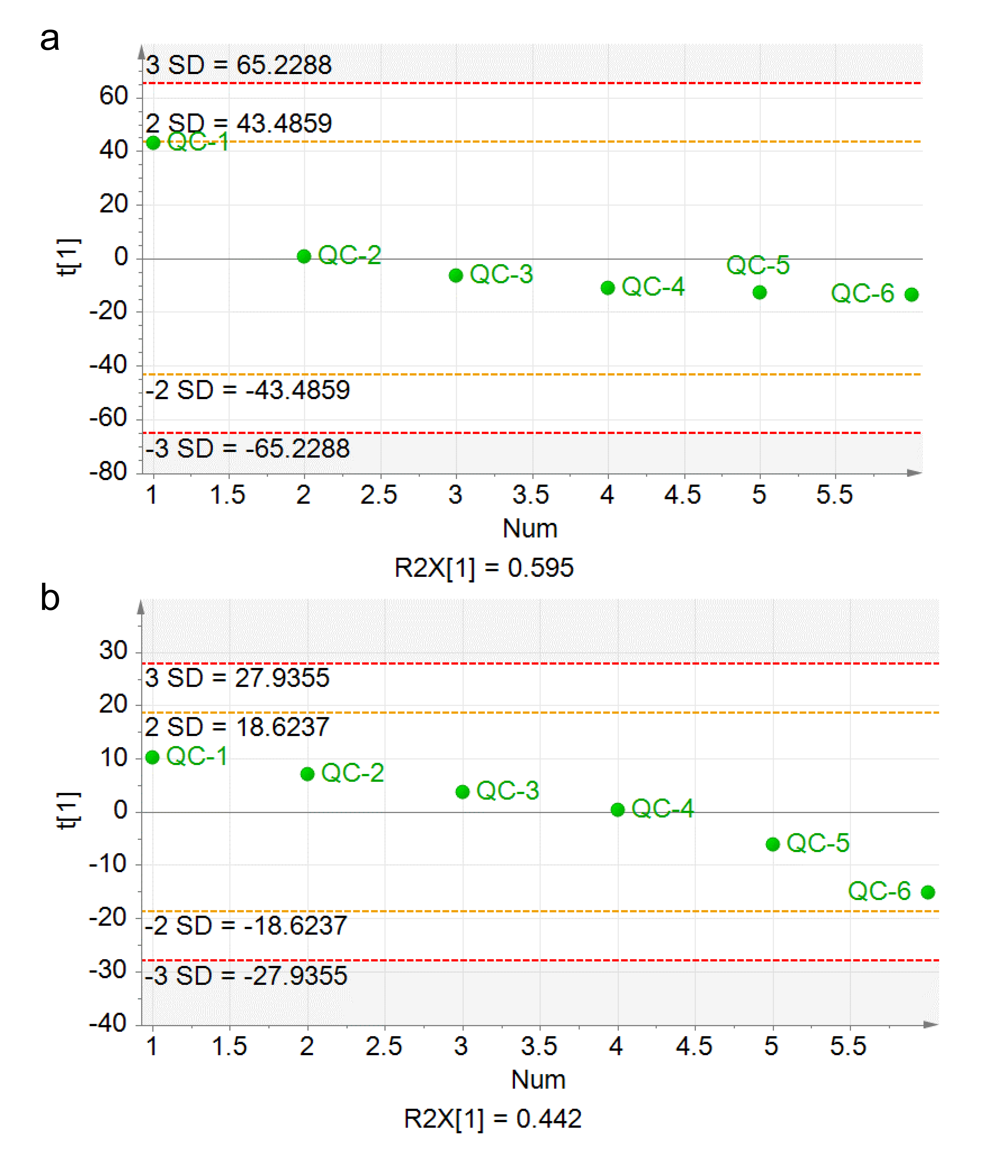

Supplement: Supplementary file 1 [file foods-11-00004-s001.zip › Figure S1.tif]

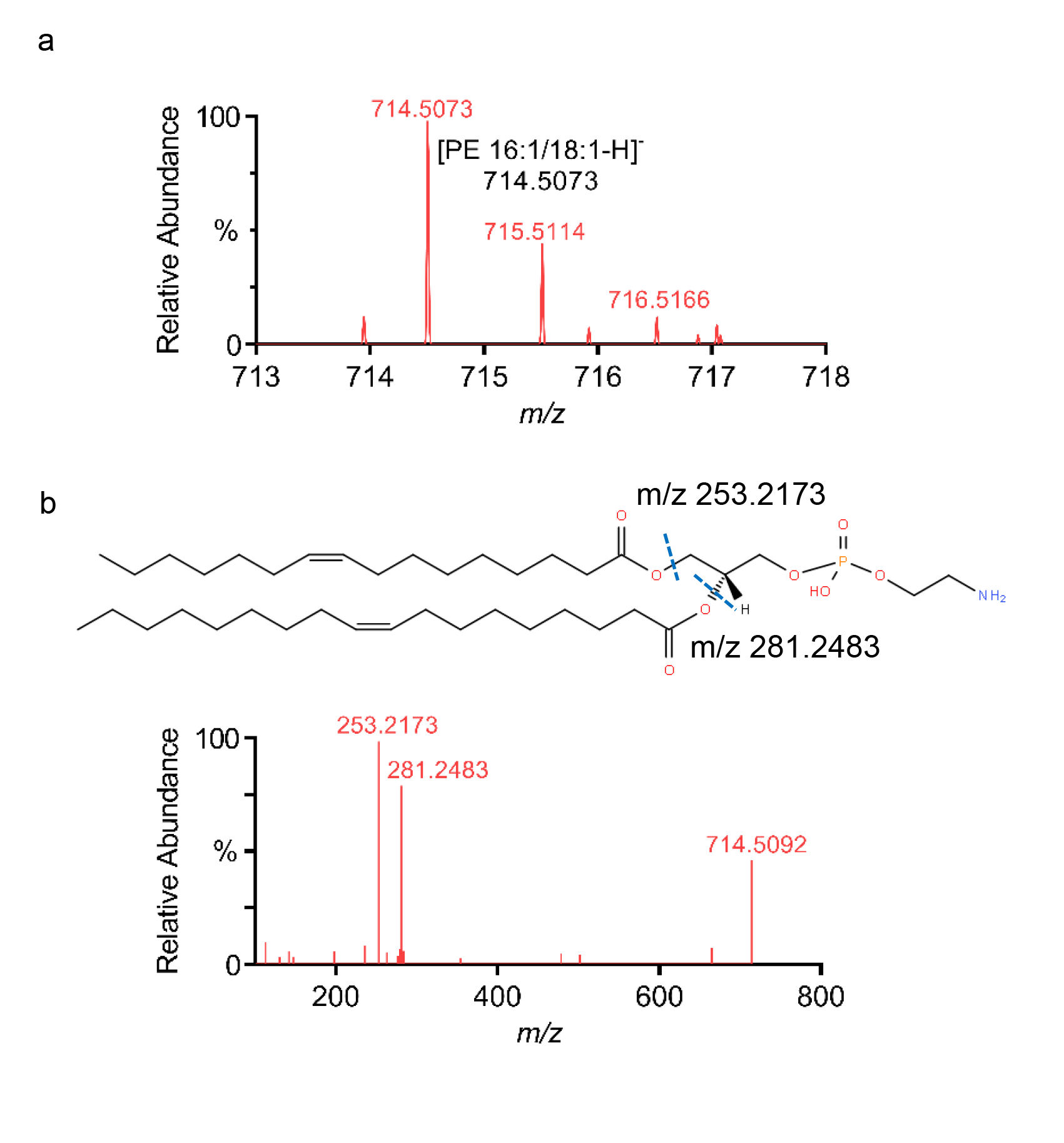

Supplement: Supplementary file 1 [file foods-11-00004-s001.zip › Figure S2.tif]
